# Proteomics in Patients with Fibromyalgia Syndrome: A Systematic Review of Observational Studies

**DOI:** 10.1007/s11916-024-01244-4

**Published:** 2024-04-23

**Authors:** Arriana Gkouvi, Sotirios G. Tsiogkas, Dimitrios P. Bogdanos, Helen Gika, Dimitrios G. Goulis, Maria G. Grammatikopoulou

**Affiliations:** 1 Unit of Immunonutrition and Clinical Nutrition, Department of Rheumatology and Clinical Immunology, Faculty of Medicine, School of Health Sciences, University of Thessaly, Biopolis, Larissa, Greece; 2 Center for Interdisciplinary Research and Innovation (CIRI-AUTH), Biomic_AUTh, Balkan Center Thermi B1.4, GR-57001 Thessaloniki, Greece; 3 Laboratory of Forensic Medicine and Toxicology, School of Medicine, Aristotle University of Thessaloniki, GR-54124 Thessaloniki, Greece; 4 Unit of Reproductive Endocrinology, 1st Department of Obstetrics and Gynecology, Medical School, Aristotle University of Thessaloniki, Thessaloniki, Greece

**Keywords:** Transferrin, Transaldolase, A2-macroglobulin, Haptoglobin, Proteome, Fibrinogen, Complement C4-A, Thrombospondin-1, Calgranulin, Hydroxytyrosol, Pain

## Abstract

**Purpose of Review:**

Fibromyalgia syndrome (FMS) is a disease of unknown pathophysiology, with the diagnosis being based on a set of clinical criteria. Proteomic analysis can provide significant biological information for the pathophysiology of the disease but may also reveal biomarkers for diagnosis or therapeutic targets. The present systematic review aims to synthesize the evidence regarding the proteome of adult patients with FMS using data from observational studies.

**Recent Findings:**

An extensive literature search was conducted in MEDLINE/PubMed, CENTRAL, and clinicaltrials.gov from inception until November 2022. The study protocol was published in OSF. Two independent reviewers evaluated the studies and extracted data. The quality of studies was assessed using the modified Newcastle–Ottawa scale adjusted for proteomic research. Ten studies fulfilled the protocol criteria, identifying 3328 proteins, 145 of which were differentially expressed among patients with FMS against controls. The proteins were identified in plasma, serum, cerebrospinal fluid, and saliva samples. The control groups included healthy individuals and patients with pain (inflammatory and non-inflammatory).

**Summary:**

The most important proteins identified involved transferrin, α-, β-, and γ-fibrinogen chains, profilin-1, transaldolase, PGAM1, apolipoprotein-C3, complement C4A and C1QC, immunoglobin parts, and acute phase reactants. Weak correlations were observed between proteins and pain sensation, or quality of life scales, apart from the association of transferrin and a2-macroglobulin with moderate-to-severe pain sensation. The quality of included studies was moderate-to-good. FMS appears to be related to protein dysregulation in the complement and coagulation cascades and the metabolism of iron. Several proteins may be dysregulated due to the excessive oxidative stress response.

**Supplementary Information:**

The online version contains supplementary material available at 10.1007/s11916-024-01244-4.

## Introduction

Fibromyalgia syndrome (FMS) is a rheumatic disease (ICD-10 M79.7) of unknown etiology, characterized by chronic widespread pain accompanied by potential neuroinflammation [1], fatigue, stress [2], memory loss, sleep disturbance, and multiple physical symptoms [3]. The prevalence of FMS in the general population ranges between 2 and 3% [4], but higher ratios have been reported in specific population groups [5]. For example, 14.8% of patients with type 2 diabetes mellitus and 80% of those with Behçet’s disease are diagnosed with FMS [4, 5]. There is however, no gold standard for the diagnostic procedure. Individuals are diagnosed based on clinical criteria suggested by the American College of Rheumatology (ACR), conceived in 1990, and revised in 2010, 2011, and 2016 [6–9]. The ACR 1990 [7] criteria rely on a clinical examination and the existence of tender points, while the 2010 [6] criteria focus on other disease parameters, including fatigue and sleep disturbances. Aside from the diagnosis, no objective biomarkers or tests have been identified to facilitate a more accurate diagnostic process or mediate the development of a precise prognostic model for FMS.

In medicine, biological markers can be used for disease detection and the discovery of drugs, as well as for monitoring the progress of patients [10]. Although numerous studies suggest plausible mechanisms driving disease development, definite evidence has been relatively scarce. FMS seems to be associated with altered central nervous system (CNS) processing, enhanced excitability, and decreased inhibition [11]. Oxidative stress, vitamin dysregulation, inflammation, autonomic dysfunction, and genetic factors may provide an insight into the pathophysiology [12–17]. Proteomics, identifying protein markers in biological fluids, can provide critical information in such complex conditions/diseases [10, 18], like FMS [19].

The present systematic review aimed to provide a comprehensive summary of the proteome of adult patients with FMS, in an effort to shed light on the pathophysiology of the condition, identify diagnostic and prognostic protein markers, and establish some potential therapeutic targets.

## Materials and Methods

### Systematic Review Protocol and PEO

The Preferred Reporting Items for Systematic Reviews and Meta-Analyses (PRISMA) [20] and the Synthesis Without Meta-analysis (SWiM) extension [21] were used. By November 2022, the study’s protocol was published at the Center for Open Science Framework (OSF) (https://shorturl.at/rHN45). The Population-Exposure-Outcome (PEO) of the research question is detailed in Supplementary Table 1.

### Search Strategy and Algorithm

Two independent reviewers (A.G. and S.G.T.) identified studies through PubMed, the Cochrane Central Register of Controlled Trials (CENTRAL), clinicaltrials.gov databases, and the grey literature from inception until November 2022. A senior reviewer (M.G.G.) resolved any discrepancies.

To identify studies in databases, we used a combination of keywords using medical subject headings (MeSH) and free text. The search was conducted in English. The keywords and search syntaxes are listed in Supplementary Tables 2 and 3, respectively.

The Rayyan [22] software was used to identify all studies fulfilling inclusion criteria and to remove duplicates. After all identified studies were imported to the software, titles and abstracts were screened to examine whether inclusion criteria were met. The remaining studies were assessed in full text.

### Inclusion and Exclusion Criteria

Studies were included in the systematic review when (1) they were observational studies on FMS, (2) of any duration, (3) in adult patients, (4) by assessing the proteome in biological fluids, (5) published until November 2022, (6) and written in the English language.

Studies were excluded when (1) they were published in another language, (2) pooling patients with FMS together with other chronic pain disorders, (3) including pediatric patients, and (4) animal or preclinical studies.

### Outcomes of Interest

Any protein identified in body fluids through proteomics in patients with FMS compared to controls was considered as an outcome of interest. Correlations of such proteins with any specific disease score or scale, like the visual analog scale (VAS) [23] for pain or the Fibromyalgia Impact Questionnaire (FIQ), were also recorded.

### Quality of Studies

The modified Newcastle–Ottawa scale (NOS) was used for assessing the quality of the included studies by two independent reviewers [24]. The maximum score a study can collect is 9 points. The scale was further adapted according to Nguyen et al. [25] and the Molecular & Cellular Proteomics (MCP) initiative [26] to fit the design and methodology of proteomic studies.

### Data Extraction

Two independent researchers (A.G. and S.G.T.) extracted data in a prespecified Excel spreadsheet. Information regarding the study (first author, year, country, funding), the sample (recruitment, number of patients and controls, age and gender of patients and controls, comorbidities, medication), the biological fluid, the fibromyalgia diagnostic criteria, the exclusion criteria and the years of diagnosis, the pain and quality of life scales VAS [23], Pressure Point Threshold (PPT) [27], tender points [28], Widespread Pain Index (WPI) [6], Symptom Severity Scale (SSS) [6], Functional Assessment of Chronic Illness Therapy (FACIT) [29], FIQ [30, 31], Pittsburgh Sleep Quality Index (PSQI) [32], Physical/Mental Component Summary-12 (PCS/MCS-12) [33], Beck Anxiety Inventory (BAI) [34], Beck Depression Inventory (BDI) [35], Hospital Anxiety and Depression Scale (HADS) [36], the methodology (proteomics methodology, database used), and the count and names of proteins were extracted for all included studies.

### Data Synthesis

Since a meta-analysis was not feasible due to the existing heterogeneity between biological samples, a systematic synthesis was conducted.

## Results

### Search Results

**Fig. 1:**
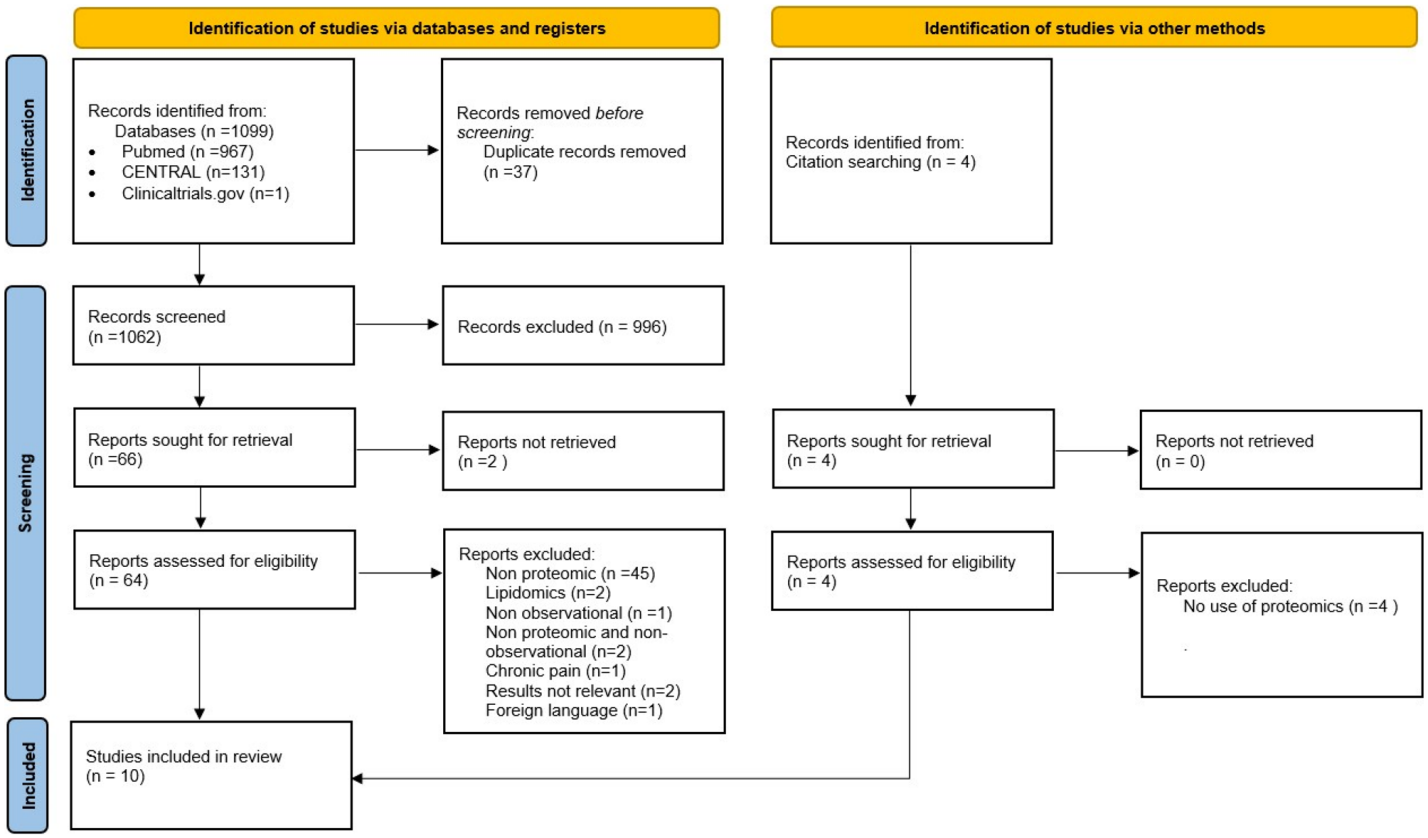
PRISMA 2020 flowchart of the studies’ selection process

Of the 1099 studies screened, 37 duplicates were removed, and 1062 were reviewed at the title and abstract level. From these, sixty-four studies were reviewed also in full-text form. Four additional studies were identified through citation searching. In total, 10 studies fulfilled the criteria (Table 1) and were included in the present systematic review [37–44, 45•, 46]. The Preferred Reporting Items for Systematic reviews and Meta-Analyses (PRISMA) 2020 [20] flowchart is presented in Fig. 1. A list of excluded studies is detailed in Supplementary Table 4.

One study was excluded for examining gut microbiome and serum metabolome in patients with FMS, combined with custom multiplex cytokine and miRNA analysis (FirePlex™ technology) in serum, thus not having relevant results to the present research question [47].

### Studies Characteristics

#### Study Design and Origin

Most studies were conducted in Europe, specifically Italy, Spain, and Sweden [37–44]. The remaining two studies were implemented in Taiwan [45•, 46]. All research was conducted between the years 2009 and 2022, with seven out of ten studies declaring funding sources [37, 38, 40, 41, 44, 45•, 46].

#### Biological Fluids

Two studies used cerebrospinal fluid (CSF) [41, 42], two saliva [38, 39], four serum samples [37, 43, 45•, 46], while the remaining two used plasma [40, 44]. One of the studies using serum samples involved metabolomics in serum and urine, with a concomitant proteome analysis in serum samples only [45•].

#### Samples

Patients were recruited through hospitals, Rheumatology and outpatient clinics, and FMS patients’ associations. The sample size ranged from 12 to 39 patients and between 12 to 90 controls, for each study. The present systematic review includes a total of 242 patients with FMS and 297 controls, the latter being either healthy, or pain controls (with inflammatory or non-inflammatory pain).

Regarding biological sex, six studies recruited female participants only [39, 40, 42–44, 46], two studies used a mixed-sex sample, with a greater percentage of women [38, 45•], one research item used female patients and mixed-sex controls [41], and the final study failed to provide data regarding participants’ sex [37] (Table 1).

**Table 1:** Characteristics and results of the included studies

		**Bazzichi** [39]	**Ciregia** [38]	**Fineschi** [37]	**Han** [46]	**Hsu** [45•]	**Khoonsari** [41]	**Khoonsari** [42]	**Ramirez-Tejero** [40]	**Ruggiero** [43]	**Wahlen** [44]
Year		2009	2018	2022	2020	2021	2018	2018	2018	2014	2020
Οrigin		Italy	Italy	Sweden	Taiwan	Taiwan	Sweden	Sweden	Spain	Italy	Sweden
Funding		NR	Yes	Yes	Yes	Yes	Yes	NR	Yes	No	Yes
Biological fluid used		Saliva	Saliva	Serum	Serum	Serum, urine	CSF	CSF	Plasma	Serum	Plasma
Recruitment site		NR	Rheumatology Unit	Health Care Center, Pain Clinic	University Hospital	Outpatient Clinics	FMS Association	Outpatient Dept of Rehabilitation	FMS Association	NR	Part of RCT
Patients (*n*)		22	30^a^	30	20	30	39	13	12	16	30
Controls (*n*)		26 HC	30 RA, 30 MG, 30 HC^a^	24 HC^b^	20 HC	25 HC	38 HC	11 RA, 8 OND	12 HC	12 HC	31 HC
Age (years)	Patients	43.4 ± 13.2	49.9 ± 12.5	48.8 ± 10.9	52.9 ± 9.6	52.1 ± 2.0	47.2 ± 9.2	47 (25–60)^f^	50.6 ± 6.3	52 ± 12	54 (13)^e^
	Controls	38.8 ± 11.5	45.4 ± 13.2 RA, 46.4 ± 14.3 MG, 42.6 ± 6.2 HC	50.0 ± 11.5	47.5 ± 7.5	51.2 ± 1.8	47.9 ± 14.4	52 (39–59)^f^ RA, 53 (45–60)^f^ OND	47.6 ± 7.9	48 ± 13	57 (9)^e^
Sex (*n*)	Patients	F	51 F, 9 M	-	F	29 F, 1 M	F	F	F	F	F
	Controls	F	52 F, 8 M RA	-	F	24 F, 1 M	5 F, 33 M	F	F	F	F
			46 F, 14 M MG 40 F, 20 M HC								
ACR criteria used		1990	NR	2009, 2016	2010	2011	1990	1990	1990	2010	1990
Duration of diagnosis		-	5.2 ± 4.3 yrs	-	9.1 ± 8.9 yrs	-	-	-	-	6.3 ± 4.5 mo	12 (14)^e^ yrs
BMI (kg/m^2^)		-	-	-	22.8 ± 4.0	-	25.8 ± 8.4	-	-	27 ± 5	26 (6)^e^
VAS		7.9 ± 1.9	6.5 ± 2.5 P	-	5.0 ± 2.2	5.3 ± 0.4	-	6.7 FM	5.6 ± 2.8	5.7 ± 3.1^c^	5.0 (3.6)^c,e^
			7.7 ± 2.5 Fa					2.5 RA			
			4.5 ± 3.1 An								
PPT (kPa)		-	-	-	230.5 ± 102	-	-	-	-	-	175 (88)^e^
Tender points (*n*)		14 ± 3	16 ± 4	-	-	-	-	-	-	-	16 (2)^e^
WPI		-	-	12.8 ± 2.6	7.4 ± 4.8	8 ± 1	-	-	-	-	-
FIQ		66.8 ± 14.4	61 ± 15	-	43.5 ± 12.7	-	-	-	52.4 ± 17.1	61.8 ± 21.9	58 (24)^e^
FACIT		-	23 ± 10	-	-	-	-	-	-	-	-
PCS-12		-	47 ± 24^d^	-	-	-	-	-	33.5 ± 9.2	-	-
MCS-12		-	48 ± 19^d^	-	-	-	-	-	38.5 ± 14	-	-
SSS		-	-	-	5.1 ± 2.5	-	-	-	-	-	-
PSQI		-	13.0 ± 3.1	-	10.4 ± 3.0	-	-	-	-	-	-
BAI		-	-	-	13.9 ± 8.8	-	-	-	-	-	-
BDI		-	-	-	14.0 ± 9.8	-	-	-	-	-	-
HADS		-	-	-	-	-	-	-	-	-	13 (14)^e^
Proteomics methodology		2-DE/MALDI-TOF/TOF	2-DE/SELDI-TOF MS	Olink proteomics	LC–MS/MS	NanoLC-MS/MS, QTOF	NanoLC-MS/MS	LC–MS/MS	NanoLC-MS/MS	2-DE/MS MS, MALDI-TOF/TOF	2-DE/MS
Database used		UniProt	-	-	UniProt	UniProt	UniProt	UniProt	UniProt	NCBI	UniProt
Identified proteins (*n*)		13	467	94	375	8	299	1422	266	3	381
Different proteins (*n*)		11	17	19	22	8	4	10	33	3	18

*2-DE* two-dimensional gel electrophoresis, *ACR* American College of Rheumatology, *An* anxiety, *BAI* Beck Anxiety Inventory [34], *BDI* Beck Depression Inventory [35], *BMI* body mass index, *CSF* cerebrospinal fluid, *F* female, *Fa* fatigue, *FACIT* Functional Assessment of Chronic Illness Therapy [29], *FIQ* Fibromyalgia Impact Questionnaire, *FMS* fibromyalgia syndrome, *HADS* Hospital Anxiety and Depression Scale [36], *HC* healthy controls, *IQR* interquartile range, *LC* liquid chromatography, *M* male, *MALDI* matrix-assisted laser desorption/ionization, *MCS-12* mental component summary [33], *MG* migraine, *MS* mass spectrometry, *NCBI* National Center for Biotechnology Information non-redundant mammalian (Taxonomy homo sapiens) database, *NOD* not other defined, *NR* not reported, *OND* other neurologic diseases, *P* pain, *PCS-12* physical component summary [33], *PPT* pressure point threshold [27], *PSQI* Pittsburgh Sleep Quality Index [32], *QTOF* quadrupole time of flight, *RA* rheumatoid arthritis, *RCT* randomized controlled trial, *SSS* symptom severity scale [6], *TOF* time of flight analyzer, *VAS* visual analog scale [23], *WPI* Widespread Pain Index [6]

^a^ Age and gender were calculated for 60 patients and 180 controls; however, proteomic analysis was conducted in 30 patients and 90 controls

^b^ One sample was discarded

^c^ Conversion to a scale 1–10

^d^ The scale is part of the SF-36 and not SF-12

^e^ Median (IQR)

^f^ Mean and range

The studies used different exclusion criteria for the selection of participants, including the diagnosis of psychiatric diseases, dementia, epilepsy, alcohol or substance abuse, hypertension, osteoarthritis, use of analgesics, autoimmune diseases, neurological diseases, diabetes mellitus, cardiovascular diseases, pregnancy or childbirth, infectious diseases, active malignant disease, use of immunosuppressants or cortisone, and history of injury. All exclusion criteria used by the studies during sample recruitment are presented in Supplementary Table 5.

#### FMS Diagnostic Criteria

For the diagnosis of FMS, the researchers utilized different diagnostic criteria in each study (Table 1), with half of the studies using the ACR 1990 [39–42, 44], two applying the ACR 2010 [43, 46], one the ACR 2016 [37], another the ACR 2011 [45•], and one not specifying which diagnostic criteria were used [38].

#### Medications

Three of the included studies listed the medications used by the included patients [37, 38, 43]. The most used drugs involved tricyclic antidepressants/amitriptyline, selective serotonin reuptake inhibitors (SSRIs) and serotonin-norepinephrine reuptake inhibitors (SNRIs), analgesics, muscle relaxants, benzodiazepines, and anticonvulsants.

#### Pain and Quality of Life Scales

Most studies used a scale to quantify patient symptomatology; however, as no common recommendations exist, the selection of such scales was arbitrary. The most frequently used scales included the VAS, which quantifies pain on a level of 1 to 10 and the FIQ, for the assessment of quality of life (QoL) [30, 31, 48, 49]. Patients’ VAS ranged from 5.0 ± 2.2 to 7.9 ± 1.9 (mean ± standard deviation), indicating that they experienced severe pain. Other scales (e.g., PPT, WPI, tender points) were used to quantify pain, while the FIQ, FACIT, PCS-12, MCS-12, and SSS were applied for the QoL [27, 33, 50]. Two research items [38, 46] studied sleep quality using the PSQI scale, with results converging on poor sleep quality, frequent sleep interruptions, and fatigue [32]. Finally, one study [46] focused on quantifying anxiety and depression using the BAI and BDI scales [35, 51], revealing an increased incidence of anxiety and depression in patients with FMS.

#### Proteomic Methodology and Database

All identified studies involved discovery proteomics, with the majority applying liquid chromatography–mass spectrometry technique for protein analysis. Liquid chromatography–mass spectrometry (LC–MS) consists of a powerful analytical technique that combines the power of LC with the highly sensitive and selective mass resolution capability of mass spectrometry (MS) [52, 53]. This technique was utilized by five of the research groups [40–42, 45•, 46]. Four research groups applied 2D electrophoresis (2-DE) techniques. Specifically, two used 2-DE coupled to matrix-assisted laser desorption ionization–time of flight mass spectrometry (MALDI-TOF MS) [39, 43], one used 2-DE/surface-enhanced laser desorption ionization–time of flight mass spectrometry (SELDI-TOF MS) [38], and one applied 2-DE/MS [44] techniques. One research item analyzed the proteins based on a targeted proteomics platform called Olink [37] which enables the analysis of some hundreds of proteins by multiplex assays.

### Quality of Included Studies

**Fig. 2:**
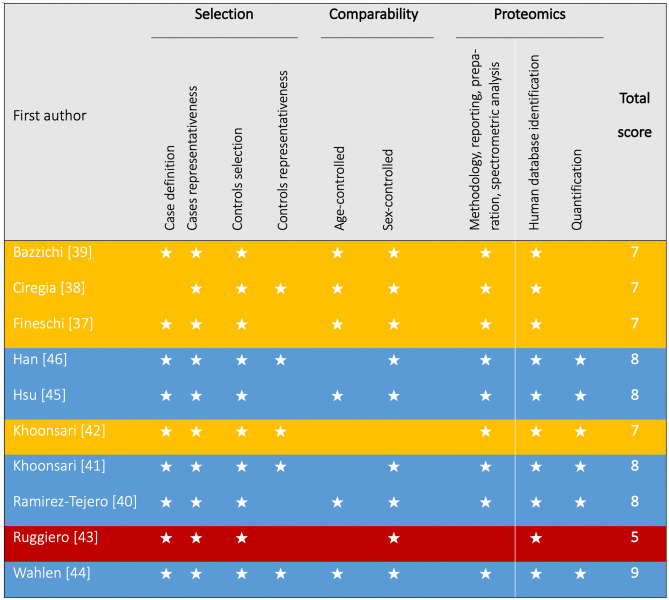
Summary results regarding studies’ quality using the modified Newcastle–Ottawa scale [24]

The summary results regarding the NOS (modified for proteomics studies) score in each included study are presented in Fig. 2. One study achieved the maximum quality score (9) [44], four studies received a total NOS score of 8 [40, 41, 45•, 46], and the remaining scored between 5 and 7 [37–39, 42, 43]. The domains with quality concerns for most studies included quantification and the representativeness of controls.

### Proteins Identified

A total of 3328 proteins were identified, including peptides and amino acids. The level of 145 proteins differed significantly between patients with FMS and controls. Based on these studies, higher number of proteins could be identified in CSF (1721 proteins from two studies), followed by plasma (647 proteins, two studies), serum (480 proteins, four studies), and saliva (480 proteins, two studies) samples.

#### Proteins Identified in Saliva and Their Role

Two studies [38, 39] assessed proteomics in saliva samples and isolated 23 differently expressed proteins in patients with FMS versus controls. One of them [39] used healthy controls, while the other [38] used a mixed sample of a healthy population, patients with rheumatoid arthritis (RA) and patients with migraine. The isolated proteins, the study in which they were identified, the UniProt ID (as the direction of change in their levels, increased or decreased relative to the healthy population) are presented in Table 2. The two studies are in agreement as they both identify transaldolase (TALDO), phosphoglycerate mutase-1 (PGAM1), and calgranulin A (S100-A8) over-expressed in patients with FMS. Additional data regarding fold change and statistical significance are provided in Supplementary Table 6.

**Table 2:** Differently expressed proteins identified in the saliva of patients with FMS versus controls, and their role

Identified proteins	UniProt ID	Difference compared to controls	Role(s)	Studies reporting difference
**TALDO**	P37837	**↑**	Pentose Pathway and NADPH [113]	Bazzichi [39], Ciregia [38]
**PGAM1**	P18669	**↑**	Glycolysis [55]	Bazzichi [39], Ciregia [38]
**S100-A8** **S100-A12**	P05109 P80511	**↑** **↑**	S100 proteins, OS [114]	Bazzichi [39], Ciregia [38]
PSMA2	P25787	↑	Ubiquitin-dependent abnormal protein degradation	Bazzichi [39]
RhoGDI2	P52566	↑	Metastasis inhibitor	Bazzichi [39]
HPR	P00739	↑	Innate immunity	Bazzichi [39]
CFL1	P23528	↑	Cytoskeleton [143]	Bazzichi [39]
CyPA	P62937	↑	Cyclosporine receptor, role in protein folding [144]	Bazzichi [39]
**PFN1**	P07737	**↑**	Cytoskeleton [73]	Bazzichi [39]
AMY1A	P04745	↑^a^	Digestion of starch in the oral cavity	Ciregia [38]
ΕΝΟ1	P06733	↑	Glycolysis, anti-ENO1 antibodies in NSI	Ciregia [38]
GSTP	P09211	↑^a^	Reduced glutathione conjugation, link to PGA2	Ciregia [38]
**IgKC**, **Iglc2**	P01834 P0CG05	↑ ↑	Constant region of immunoglobulin light chains	Ciregia [38]
pIgR	P01833	↓^a^	IgA and IgM transcytosis in mucosal epithelial cells	Ciregia [38]
Albumin	P02768	↑^b^	Plasma protein	Ciregia [38]
**TRFE**	P02787	**↑**	Main iron-binding protein [82]	Ciregia [38]
CYS	P01037	↓	Cell cycle	Ciregia [38]

Proteins appearing in more than one study are presented in bold font. ↑ Increased, ↓ decreased

*AMY1A* α-amylase 1, *CFL1* cofilin-1, *CNS* central nervous system, *CyPA* cyclophilin A, *CYS* cystatin, *ΕΝΟ1* α-enolase, *FMS* fibromyalgia syndrome, *GSTP* glutathione S-transferase P, *HPR* haptoglobin-related protein precursor, *Iglc2* Ig λ-2 chain C regions, *IgKC* Ig kappa chain C region, *S100-A8* calgranulin Α, *S100-A12* calgranulin C, *NADPH* nicotinamide adenine dinucleotide phosphate non-oxidized, *NSI* connective tissue diseases, *OS* oxidative stress, *PFN1* profilin-1, *PGA2* prostagladin A2, *pIgR* polymeric immunoglobulin receptor, *PSMA2* proteasome subunit-α-type-2, *RhoGDI2* Rho GDP-dissociation inhibitor 2, *TALDO* transaldolase, *TRFE* transferrin

^a^↑ vs healthy controls

^b^↑ vs healthy controls and migraine, but ↓ vs rheumatoid arthritis

TALDO mediates cell shape changes and cell motility and is part of the pentose pathway, associated with NADPH production. As a result, its high levels could be interpreted as the body’s attempt to increase NADPH production to deal with oxidative stress [54]. PGAM1 is an enzyme of the glycolysis pathway, and its elevated levels link FMS to glucose disorders. Additionally, anti-PGAM1 antibodies have been observed in various autoimmune diseases of the CNS [55]. Finally, calgranulin A is a low molecular weight protein that binds calcium and belongs to the S100 protein family, regulating various intracellular processes. An increase in the concentration of calgranulin A is probably associated with oxidative stress [56].

#### Proteins Identified in the CSF and Their Role

Two different studies published by the same author group performed CSF proteomics [41, 42]. A total of 14 different proteins were identified as being over- or under-expressed in patients with FMS, compared to controls, as shown in Table 3. Additional details are presented in Supplementary Table 7. Proteins identified in more than one study included apolipoprotein C-III, which was also isolated in plasma at low concentrations [44], and complement C4-A, which was observed in high levels at the serum of patients with FMS [46].

**Table 3:** Differently expressed proteins identified in the CSF of patients with FMS versus controls and their role

Identified proteins	UniProt ID	Difference compared to controls	Role(s)	Studies reporting difference
L1CAM	P32004	↑	Neuronal structure–function, synaptic plasticity [145]	Khoonsari [42]
**C4-A**	P0C0L4	**↑**	Classical complement pathway [146]	Khoonsari [42]
LYSC	P61626	↑	Bacteriolytic	Khoonsari [42]
PTPRz	P23471	↑	Differentiation into mature, fully myelinated oligodendrocytes, protection from apoptosis [147]	Khoonsari [42]
ApoD	P05090	↓	Component of HDL	Khoonsari [41]
α1ACT	P01011	↓	Unclear	Khoonsari [42]
PGRN	P28799	↑	Regulator of lysosomal function and growth factor involved in inflammation and cell proliferation [148]	Khoonsari [42]
LRP1	Q07954	↓^a^	Phagocytosis of apoptotic cells [149]	Khoonsari [42]
**ApoC3**	P02656	**↑**	Atherogenic protein [57]	Khoonsari [41]
LG3BP	Q08380	↑	Cell-to-cell adhesion, pro-inflammatory signaling	Khoonsari [41]
PCSK1	P29120	↑	Neuropeptides [150]	Khoonsari [41]
MDH	P40925	↓	Gluconeogenesis [60]	Khoonsari [41]
CAMKIIα	Unknown	↓^a^	Many cascades, learning and memory	Khoonsari [42]
cKIT	A0A0U2N547	↑^b^	Stem cell growth factor receptor	Khoonsari [42]

Proteins appearing in more than one study are presented in bold font. ↑ Increased; ↓ decreased

*α1ACT* Α1 antichymothrypsin, *ApoC3* apolipoprotein C-III, *ApoD* apolipoprotein D, *C4-A* complement C4-A, *CAMKIIα* calcium/calmodulin-dependent protein kinase type II subunit α, *FMS* fibromyalgia syndrome, *HDL* high-density lipoprotein, *LG3BP* galectin-3-binding protein, *LRP1* low-density lipoprotein receptor-related protein 1, *L1CAM* L1 cell adhesion molecule, *LYSC* lysozyme C, *MDH* malate dehydrogenase, *PCSK1* proprotein convertase subtilisin/kexin type 1, *PGRN* progranulin, *PTPRz* receptor-type tyrosine-protein phosphatase zeta

^a^Decrease relative to other neurologic diseases, increase relative to rheumatoid arthritis

^b^Relative to rheumatoid arthritis

Apolipoprotein C-III is an atherogenic protein inhibiting lipoprotein lipase (LPL) activity, displacing LPL from lipid particles. Furthermore, it acts in an inhibitory manner at the triglyceride cycle, leading to higher triglyceride concentrations, which result in arterial stiffness and an increased risk of cardiovascular events [57]. Isolation of apolipoprotein C-III in the CSF has been described in the literature; however, its role in the CNS remains unclear [58]. Complement C4-A is part of the classical complement pathway and is involved in forming the membrane attack complex (MAC), activated by immune complexes and leading to cell lysis [59].

Three additional proteins may play a role in the pathogenesis of FMS: malate dehydrogenase, galectin-binding protein-3 (LG3BP), and ProSAAS (PCKS1). Malate dehydrogenase is involved in gluconeogenesis and energy production for muscle contraction. Changes in protein levels related to glycolysis and gluconeogenesis were also observed in the saliva of patients with FMS [60]. Decreased malate dehydrogenase levels result in low malate concentration; thus, a “Super Malic” dietary supplement has been suggested to have beneficial effects in tampering down FMS symptomatology [61]. It is also worth mentioning that lower levels of malate dehydrogenase have been observed in the synovial fluid of patients with RA, reflecting disturbances in its metabolism [61]. LG3BP is a regulator of pro-inflammatory signaling and is elevated in patients with RA, affecting the balance between pro-inflammatory and anti-inflammatory processes [62]. Finally, PCKS1 is present in the neurons, including processing fetal neuropeptides and inhibiting the enzyme that converts prohormones to their active form [63, 64]. PCKS1 is proteolytically processed and produces various neuropeptides [65]. Thus, elevated PCKS1 concentrations may reflect endocrine disturbances already reported in the literature among patients with FMS [66].

#### Proteins Identified in Plasma and Their Role

Serum and plasma were studied separately, as they are different biological materials. Plasma is the blood derivative obtained by adding anticoagulant and centrifugation, while serum is the blood derivative that remains after coagulation for 30 min and centrifugation. As a result, differences arise in proteomic and metabolomic studies depending on the biological fluid studied [67]. Two research groups assessed plasma proteomics and reported identifying four common proteins at different levels compared to controls, namely transferrin (TRFE), haptoglobin, α2-macroglobulin, and fibrinogen β chain. TRFE has also been observed at elevated levels in the saliva [38] of patients with FMS. The proteins isolated in plasma and their role are shown in Table 4, while additional details can be found in Supplementary Table 8.

**Table 4:** Differently expressed proteins identified in the plasma of patients with FMS versus controls and their role

Identified proteins			UniProt ID	Difference compared to controls	Role(s)	Studies reporting difference
Complement		**C1Qc** C9 C2 CFAΗ C1S C7 C1r C3B CFI C4B	P02747 P02748 P06681 P08603 P09871 P10643 P00736 P01024 P05156 P0C0L5	**↑** ↑ ↑ ↑ ↑ ↑ ↓ ↔ ↔ ↔	Apart from CFAH (an inhibitory protein of the alternative pathway), the remaining are involved in forming the membrane attack complex. This pathway is triggered by IgM/IgG-immunocomplexes, leading to chemotaxis and plasma protein secretion, facilitating opsonization. C3B acts as an opsonin [146]	Ramirez-Tejero [40] Wahlen [44]
Coagulation		IX, Χ, **FGA**, **FGB**, **FGG**, protein S, ZPI, PLG, KNG1, B2GP, α2AP	P00740 P00742 P02671 P02675 P02679 P07225 Q9UK55 P00747 P01042 P02749 P08697	↑ **↑** ↓ ↑ ↑ ↓ ↑ ↓ ↓ ↑ ↔	The complement cascade can be activated by coagulation proteins. FGA, FGB, and FGG form fibrin. IX and X are activators of the complement cascade. ZPI is an anticoagulant protein that inhibits the activity of clotting factors. PLG is converted to plasmin, while B2GP is associated with the APS [151]	Ramirez-Tejero [40] Wahlen [44]
Lipid metabolism:		ApoL1, ApoC2, **ApoC3**, FETUB	O14791 P02655 P02656 Q9UGM5	↑ ↑ ↓ ↓	ApoL1 is part of HDL; ApoC2 and ApoC3 regulate triglyceride metabolism. Relationship of FETUB to atherosclerosis [152]	Ramirez-Tejero [40] Wahlen [44]
Ig structure		IgHM, IgAC, **IgKC**	P01871 P01876 P01834	↑ ↑ ↓	Parts of Igs, each with specific roles in the immune response	Ramirez-Tejero [40] Wahlen [44]
Acute phase reactants		**SAP,** **SAA4** A1AGP1, A1AGP2, **Hp**, HRG, PZP	P02743 P35542 P02763 P19652 P00738 P04196 P20742	**↑** **↑** ↑ ↑ **↑** ↓ ↑	Inflammation and tissue damage response. Concentration increases in acute phase reactions. HRG is a negative inflammation marker, with levels decreasing in the acute phase [153]	Ramirez-Tejero [40] Wahlen [44]
Hormone-related		TBG, CBG, AGT	P05543 P08185 P01019	↓ ↓ ↓	TBG and CBG are thyroxine and corticosteroid transporters, respectively AGT is linked to the RAAS	Ramirez-Tejero [40]
**Transferrin (TRFE)**			P02787	**↑**	Iron-binding protein [68]	Ramirez-Tejero [40] Wahlen [44]
**A2M**			P01023	↔	Protease inhibitor, immune response (trapping proteases), coagulation, inflammation, autoimmunity [135]	Ramirez-Tejero [40] Wahlen [44]
CA			P00915	↓	Acid–base balance	Ramirez-Tejero [40]
CD14			P08571	↓	Recognizes bacterial components, immune response [154]	Ramirez-Tejero [40]
Structural	**GSN**, Aktin cytoplasmic 1		P06396 P60709	↑ ↑	Cytoskeleton	Ramirez-Tejero [40] Wahlen [44]
**THBS1**			P07996	↑	Angiogenesis inhibition, coagulation cascade [79]	Ramirez-Tejero [40]
A1BG			P04217	↓	Unknown [155]	Wahlen [44]

Proteins appearing in more than one study are presented in bold font. ↑ Increased; ↓ decreased; ↔ unclear result

*A1AGP1* Alpha-1-acid glycoprotein 1, *A1AGP2* Alpha-1-acid glycoprotein 2, *A1BG* A-1B-glucoprotein, *A2AP* α2-antiplasmin, *A2M* Α2-macroglobulin, *AGT* angiotensinogen, *ApoC2* apolipoprotein C2, *ApoC3* apolipoprotein, *ApoL1* apolipoprotein L1, *APS* anti-phospholipid syndrome, *B2GP* Β2-glycoprotein, *C1Qc* complement C1q subcomponent subunit C, *C1r* complement C1r subcomponent, *C1s* complement C1s subcomponent, *C2* complement C2, *C3B* complement C3 beta chain, *C4B* complement C4 beta chain, *C7* complement C7, *C9* complement C9, *CA* carbonic anhydrase, *CBG* corticosteroid-binding globulin, *CD14* cluster of differentiation 14, *CFAH* complement factor H, *CFI* complement factor I light chain, *FETUB* fetuin-B, *FGA* fibrinogen α, *FGB* fibrinogen β, *FGG* fibrinogen γ, *FMS* fibromyalgia syndrome, *GSN* gelsolin, *HDL* high-density lipoprotein, *Hp* haptoglobin, *HRG* histidine-rich glycoprotein, *Ig* immunoglobulin, *IgAC* Ig alpha-1 chain C region, *IgHM* Ig mu chain C region, *IgKC* Ig kappa chain C region, *IX* coagulation factor IX, *KNG1*, kininogen-1, *PLG* plasminogen, *PZP* pregnancy zone protein, *RAAS* renin–angiotensin–aldosterone system, *SAA4* serum amyloid A4 component, *SAP* serum amyloid P component, *TBG* Thyroxine-binding globulin, *THBS1* thrombospondin-1, *X* coagulation factor X, *ZPI* protein Z-dependent protease inhibitor

TRFE is the main iron-binding protein. In the inflammatory context of chronic lung disease, the increase in TRFE may reflect an attempt to avoid the deleterious effects of free iron [68]. By binding the free iron, the body is protected from potential damage. Haptoglobin is an acute phase marker and binds to hemoglobin during hemolysis to limit the oxidative properties of heme and allow the complex to be recognized by the macrophage receptor CD163 [69]. It protects the organism from oxidative stress. On the other hand, α2-macroglobulin consists of a protease inhibitor, participating in the immune response by trapping proteases, while it is also involved in the coagulation cascade by inhibiting the anticoagulant effect of protein S [70]. Fibrinogen α, β, and γ chains polymerize and form fibrin, the basic component of the clot. These proteins were isolated in serum and plasma [45•, 46] samples of patients with FMS.

Several isolated proteins are associated with the acute phase reaction, the complement cascade, and coagulation-fibrinolysis. Increased concentrations of TNF-α, IL-8, and IL-10 in FMS have been reported in the literature; however, the data are conflicting as other studies have not confirmed them [16, 71].

#### Proteins Identified in the Serum and Their Role

Serum was studied by four research teams (Table 5, Supplementary Table 9) [37, 43, 45•, 46]. The proteins identified in the serum of patients with FMS that differed from controls and were reported by most studies included fibrinogen A chain and profilin-1 (Table 5). Proteins identified in the serum and plasma involved A, B, and C chain of fibrinogen, serum amyloid (P-component, protein A4), C1qC, and thrombospondin-1. Calgranulin C and the immunoglobin Ig lambda-2 chain C region were identified in both the serum and saliva of patients with FMS. Finally, a common protein between serum and CSF was complement C4-A.

**Table 5:** Differently expressed proteins identified in the serum of patients with FMS versus controls and their role

Identified proteins		UniProt ID	Difference compared to controls	Role(s)	Studies reporting difference
Complement	**C4A**, **C1qC**	P0C0L4 P02747	**↑** **↑**	Classical complement pathway [59, 156]	Han [46] Hsu [45•]
Coagulation	**FGA,** **FGB,** **FGG,** Prothrombin, GPV, GP1BA	P02671 P02675 P02679 P00734 P40197 P07359	**↓** **↓** **↓** ↓ ↓ ↓	FGA, FGB, and FGG polymerize and create fibrin. Platelet glycoproteins allow platelets to adhere to damaged vessel walls [151]	Han [46] Hsu [45•]
Retinoid transporters:	TTR, RBP4	P02766 P02753	↑ ↑	The increase might be due to hypovitaminosis A [43]	Ruggiero [43]
Ig form:	GLV3-25, HG3, GHV1OR15-1 **IGLC2**	P01717 P01743 P23083 P0CG05	↓ ↓ ↓ **↓**	Parts of Igs, each with a specific role in immune response	Han [46]
Acute phase	**SAP,** **SAA4**	P02743 P35542	**↑** **↓**	Inflammation response, tissue damage. Levels increase in acute phase reactions [153]	Han [46]
Cell signaling, cytokines, receptors	IL1RAP, CD16B, CD16A, IL-18R1, CD40, IL-10RB, TNFRSF9, IL-8, TNF, TNFSF14, CD5, CCL3	Q9NPH3 O75015 P08637 Q13478 P25942 Q08334 Q07011 P10145 P01375 O43557 P06127 P10147	↑ ↑ ↑ ↑ ↑ ↑ ↑ ↑ ↑ ↑ ↑ ↑	Inflammatory: IL1RAP, IL-8, TNF, TNFSF14, CCL3 Anti-inflammatory: IL-10RB Regulatory: IL-18R1, CD5 Pro-inflammatory, depending on signaling: TNFRSF9 CD16B and CD16A are NK cell receptors binding to the Fc region, participating in antibody-dependent cellular cytotoxicity [157, 158]	Fineschi [37] Han [46]
Growth factors	FGF-21, FGF-19, FGF-23, CSF-1	Q9NSA1 O95750 Q9GZV9 P09603	↑ ↑ ↑ ↑	FGF/FGFR pathways participate in the development of most organs, angiogenesis and lymphangiogenesis [159]	Fineschi [37]
KRT80, **PFN1**		Q6KB66 P07737	↑ **↓**	Cytoskeleton. PFN1 interacts with autophagy proteins [72]	Han [46]
Enzymes	SIRT2, SULT1A1, MMP10, HMT1	Q8IXJ6 P50225 P09238 O95568	↑ ↑ ↑ ↑	SIRT2, a cell-cycle regulator, deacetylates target proteins. SULT1A1 transfers a sulfur group. MMP10 degrades extracellular substance components, HMT1 is a methyltransferase	Fineschi [37] Han [46]
Protease inhibitors	AAT, SEPRINB3	P01009 P29508	↑ ↓	Protease inhibition, tissue protection. AAT protects lungs from neutrophil elastase [160]	Hsu [45•] Ruggiero [43]
S100	S100-A7, **S100-A12**	P31151 P80511	↓ **↑**	A12 is related to OS [56]. A7 is related to psoriasis [161]	Fineschi [37] Hsu [45•]
**THBS1** THBS2		P07996 P35442	**↓** ↓	Inhibition of angiogenesis, coagulation [79]	Han [46]
Skin	LYVE1, Gal-7, hornerin	Q9Y5Y7 P47929 Q86YZ3	↑ ↓ ↑	Skin role, LYVE1 is the most specific lymphedema marker [162]	Han [46] Hsu [45•]
STAMBP, Axin-1, TMPRSS13		O95630 O15169 Q9BYE2	↑ ↑ ↑	Transport	Fineschi [37] Han [46]

Proteins found elevated in ≥ 2 studies in patients with FMS compared to the controls are shown in bold fonts

*AAT* Alpha1-antitrypsin, *C4A* complement C4A, *CCL3* chemokine ligand 3, *CD16A* low affinity immunoglobulin gamma Fc region receptor III-A, *CD16B* low affinity immunoglobulin gamma Fc region receptor III-B, *CD5* T-cell surface glycoprotein CD5, *FGA*, *FGB*, *FGG* fibrinogen α, β, γ chains, *FGF/FGFR* fibroblast growth factor/FGF receptor, *FMS* fibromyalgia syndrome, *Gal-7* galectin 7, *GHV1OR15-1* Ig heavy chain V-I region V35, *GLV3-25* Ig lambda chain V-IV region Hil, *GPV* glycoprotein V, *GP1BA* platelet glycoprotein Ib alpha chain, *HG3* Ig heavy chain V-I region HG3, *HMT1* histidine protein methyltransferase 1 homolog, *Ig* immunoglοbulin, *Iglc2* Ig lambda-2 chain C region, *IL-8* Interleukin-8, *IL1RAP* Interleukin-1 receptor accessory protein, *IL-18R1* Interleukin-18 receptor 1, *IL-10RB* Interleukin-10 receptor B, *KRT80* keratin type II cytoskeletal 80, *LYVE1* lymphatic vessel endothelial hyaluronan receptor 1, *MMP10* stromelysin-2, *NK* natural killer, *OS* oxidative stress, *PFN1* profilin-1, *RBP4* retinol binding protein 4, *ROS* reactive oxygen species, *S100-A7* psoriasin, *S100-A12* calgranulin C, *SAA4* serum amyloid A4 component, *SAP* serum amyloid P component, *SERPINB3* serpin B3, *SIRT2* sirtuin 2, *STAMBP* STAM-binding protein, *SULT1A1* sulfotransferase 1Α1, *THBS1* thrombospondin-1, *THBS2* thrombospondin-2, TNF tumor necrosis factor, *TNFRSF9* TNF Receptor Superfamily Member 9, *TNFSF14* TNF Receptor Superfamily Member 14, *TMPRSS13* transmembrane protease serine 13, *TTR* transthyretin

Profilin-1 is a protein that regulates actin dynamics in cells by promoting actin filament assembly and turnover, essential for processes like cell motility, shape maintenance, and endocytosis. It is crucial in facilitating cellular movement and maintaining cell structure [72, 73]. The serum amyloid p-component reduces neutrophil adhesion to proteins, inhibits the differentiation of monocytes into fibrocytes, attenuates profibrotic macrophages, activates the complement pathway, and promotes phagocytosis of cell debris. These effects regulate key aspects of inflammation and set a threshold for immune cell activation [74–76]. Serum amyloid A4 is an acute phase reactant with a procoagulant role [77]. Finally, thrombospondins have diverse tissue-specific actions that include effects on angiogenesis, platelet activation, inflammation, and cell death that directly impact wound healing and tumorigenesis [78, 79].

#### Proteins Identified in Several Biological Fluids

More than one study identified 20 proteins differentially expressed in patients with FMS compared to controls. Specifically, fibrinogen α, β, and γ chains were identified in serum and plasma samples. These proteins play a key role in the coagulation cascade, polymerize, and create fibrin. However, their levels varied between studies. TRFE, the major iron-binding protein, was elevated in plasma and saliva samples, while profilin-1 was detected in three studies with decreased concentrations in serum and higher levels in saliva samples of patients with FMS. Interestingly, one investigator [45•] characterized profilin-1 as a discriminating marker between two subgroups of FMS, experiencing pain and stiffness as prominent symptoms, respectively.

C4-A concentrations were elevated in serum and CSF samples, and C1qC was observed in high levels in the plasma and serum samples of patients with FMS. A2-macroglobulin and serum amyloid (A4 and p-component) were isolated in plasma and serum, while haptoglobin was identified in plasma samples. Similarly, thrombospondin-1 was identified in plasma and serum samples. Elevated PGAM1, TALDO, and calgranulin A levels were observed in saliva samples of patients with FMS. Calgranulin C was over-expressed in serum and saliva of patients with FMS, while several immunoglobulin fractions were isolated in plasma, serum, and saliva samples. All proteins identified as being differentially expressed in FMS compared to controls in more than one study are presented in Table 6.

**Table 6:** Proteins identified in patients with FMS compared to controls, by more than one study

Identified proteins	Uniprot ID	Levels in FMS biological fluids compared to controls				Studies reporting difference (*n*)
		Serum	Plasma	Saliva	CSF	
FGA	P02671	↓^a^	↓^a^			3 [44, 45•, 46]
FGB	P02675	↓	↑			3 [40, 44, 45•]
PFN1	P07737	↓		↑		3 [39, 45•, 46]
TRFE	P02787		↑	↑		3 [38, 40, 44]
Α2Μ	P01023		↑^b^			2 [40, 44]
ApoC3	P02656		↓		↑	2 [41, 44]
Complement C4A	P0C0L4	↑			↑	2 [42, 46]
Complement C1qC	P02747	↑	↑			2 [40, 45•]
FGG	P02679	↓	↑			2 [40, 45•]
Hp	P00738		↑			2 [40, 44]
Ig λ-2 chain C regions	P0CG05	↓		↑		2 [38, 46]
Ig kappa chain C region	P01834		↓	↑		2 [38, 44]
Ig α-1 chain C region	P01876		↑	↑		2 [38, 40]
PGAM1	P18669			↑		2 [38, 39]
SAA4	P35542	↓	↑			2 [40, 46]
SAP	P02743	↑	↑			2 [40, 46]
S100-A12	P80511	↑		↑		2 [37, 39]
S100-A8	P05109			↑		2 [38, 39]
TALDO	P37837			↑		2 [38, 39]
THBS1	P07996	↓	↑			2 [40, 46]

*A2M* Α2-macroglobulin, *ApoC3* apolipoprotein C-III, *FGA* fibrinogen A chain, *FGB* fibrinogen β chain, *FGG* fibrinogen γ chain, *FMS* fibromyalgia syndrome, *Hp* haptoglobin, *Ig* immunoglobulin, *PGAM1* phosphoglycerate mutase 1, *PFN1* profilin-1, *S100-A8* calgranulin Α, *S100-A12* calgranulin C, *SAA4* serum amyloid Α4, *SAP* serum amyloid P-component, *TALDO* transaldolase, *TRFE* transferrin, *THBS1* thrombospondin-1

^a^One study failed to mention levels

^b^Decreased in one study

### Association of Proteins with Scales of Disease Severity and Quality of Life

Bazzichi et al. [39] assessed the association between elevated saliva TALDO and PGAM1 levels and pain (VAS), QoL (FIQ), and the number of tender points. However, no significant association was detected. Similarly, Ciregia et al. [38] failed to observe any association between isolated proteins in saliva and pain (VAS), QoL (FIQ, FACIT), or tender points count.

Han et al. [46] found a negative correlation between serum keratin (Keratin, type II cytoskeletal 80) and depressive symptoms (using the BDI scale) (*P* = 0.014, *r* =  − 0.567) and a mild correlation between the GHV1-46 immunoglobulin segment (Ig heavy chain V-I region HG3) and pain (using the VAS scale, *P* = 0.049, *r* = 0.470).

Wahlen et al. [44] correlated elevated α2-macroglobulin and plasma TRFE concentrations to moderate-to-severe pain intensity. On the other hand, Ruggiero et al. [43] failed to report any associations between isolated serum proteins, disease duration, and pain (VAS) and QoL (FIQ) scales. Finally, Hsu et al. [45•] attempted to discriminate pain and soreness phenotypes in patients with FMS into two groups, focusing on the predominant reported symptom (pain or stiffness), concluding that this allocation is possible using proteomics, as each group exhibits different concentrations of proteomic markers.

## Discussion

The results presented herein reveal that the identified proteins with significant alterations in the biological fluids of patients with FMS are mainly related to the immune system, the complement cascade, coagulation, and fibrinolysis.

### Iron Metabolism Biomarkers

The most prevalent protein isolated in the included studies involved TRFE, over-expressed in plasma and saliva in FMS compared to healthy and pain controls. Since TRFE is an iron-binding protein, it reflects the body’s need for iron supply [80]. The association of FMS and iron metabolism disorders has been reported, with a possible mechanism being the involvement of iron in the production of dopamine and serotonin as a cofactor [81]. Elevated TRFE concentrations have been observed in the CSF of patients with restless legs syndrome [82, 83]. However, in a case–control study, no difference was observed between patients with FMS and controls regarding serum iron, TRFE, and ferritin levels [84]. Recently, a Taiwan nationwide study [85] revealed that adults with iron deficiency anemia had increased chances of developing FMS. An additional piece in the puzzle was provided by an intervention study [86] conducted on women with FMS and low ferritin levels, who had previously failed to improve iron status with oral supplements. When intravenous iron (ferric carboxymaltose) treatment was initiated, a reduction in pain sensation and an improvement in QoL were noted [86]. Similar results were also reported from a blind, randomized, placebo-controlled trial [87], indicating a role for iron, in FMS treatment among patients with low iron stores.

### Coagulation Biomarkers

Concentrations of fibrinogen α, β, and γ chains differed between patients and controls in serum and plasma samples, but findings regarding the direction of difference varied among investigators. Research is consistent with the elevated fibrinogen levels among patients with FMS, indicative of a prothrombotic state [88]. Complement C1QC and C4A were also elevated in various biological fluids (CSF, plasma, serum) in FMS. These proteins are involved in the formation of MAC through the classical complement pathway, which is stimulated by IgM/IgG immune complexes and leads to chemotaxis and mobilization of cell opsonization [59, 89]. Certain coagulation proteins can also activate the complement cascade [90]. These findings are consistent with the involvement of the coagulation cascade and complement in the pathophysiology of FMS.

### Inflammation Biomarkers

Inflammation is a known driver of FMS. The increased amounts of skin mast cells [91, 92], substance P, and corticotropin-releasing hormone (CRH) have been observed in FMS [93, 94], appear to activate the release of IL-8 and monocyte chemoattractant protein-1 (MCP-1, a pro-inflammatory chemokine, member of a subfamily of the IL-8 supergene family) [95], elevating plasma concentrations [96]. As a result, both IL-8 and MCP-1 have been suggested as possible diagnostic biomarkers for FMS, conferring an inflammatory action [97]. At the proteomics level, different concentrations of thrombospondin-1 were observed in the serum and plasma of patients with FMS compared to controls. Inflammation in the CNS has been suggested as a mechanism involved in the pathogenesis of FMS, as impaired coagulation and fibrinolysis have been associated with degeneration of the CNS [98]. The processing of pain is transmitted from peripheral tissues to the brain and is influenced by a variety of endogenous and exogenous processes [96], including impaired coagulation and fibrinolysis [98]. Disturbances in any point in these pathways have been also observed in proteomic studies among patients with multiple sclerosis [99]. In parallel, small-fiber neuropathy (SFN) has been an additional consistent finding in FMS [100–102], indicative of CNS degeneration. However, more research is required to clarify the association between neuroinflammation and FMS.

As for the possible therapeutic component of anti-inflammatory regimes, research has revealed that the consumption of refined olive oil (ROO) among patients with FMS reduced fibrinogen levels, platelet distribution width, neutrophil-to-lymphocyte ratio, and erythrocyte sedimentation rate (ESR) concentrations [103].

### Oxidative Stress Biomarkers

Haptoglobin is an acute phase protein with antioxidant activity that binds hemoglobin and prevents the toxic effect of iron. Elevated haptoglobin levels were recorded in the plasma of patients with FMS as a possible coping mechanism to mitigate oxidative stress [104]. Conventional analytical methods also verify the existence of higher plasma haptoglobin levels in FMS and associate them with symptoms of depression, hyperalgesia, exhaustion, and sleep disturbances [105]. Recent research suggests increased oxygen free radicals among patients with FMS [106]. In more detail, prooxidative factors such as nitric oxide, products of free radical lipid peroxidation, including serum malondialdehyde and lipid hydroperoxide demonstrate an increased concentration, whereas xanthine oxidase levels are decreased, a process commonly occurring when oxygen free radicals are produced [106–109]. In parallel, the levels of endogenous antioxidants are reduced, including glutathione, superoxide dismutase, and total antioxidant status in serum, as a response to the elevated oxidative stress [106, 110]. This phenomena have led to several researchers arguing whether FMS is actually an oxidative stress–driven disorder [107], since greater levels of prooxidative factors and mitophagy appear to augment pain sensitization [109]. Interestingly, a recent systematic review concluded that supplementation with antioxidant vitamins and coenzyme Q10 for at least 6 weeks was associated with a reduced pain perception in 80% of the patients with FMS [111], indicating that antioxidants appear to have an analgesic role in FMS management [111, 112].

TALDO is an enzyme of the pentose pathway that leads to NADPH production [54, 113]. Over-expression of TALDO was recorded in the saliva of patients with FMS, and this increase may also reflect the body’s attempt to compensate for the increased oxidative stress endured. Although higher TALDO levels were observed in FMS compared with the healthy population, no differences were noted between FMS and patients with RA, or migraine.

Compared to healthy controls, patients with FMS demonstrated increased calgranulin A (S100-A8), but when compared to patients with RA and migraine, this difference ceased to exist. On the other hand, calgranulin C (S100-A12) concentrations were elevated in the saliva and serum of patients with FMS. Calgranulins are homogeneous low molecular weight calcium-binding proteins belonging to the S100 protein family. They are involved in various cellular responses and intracellular pathways that regulate cell differentiation, cytoskeleton, structural organization of membranes, intracellular calcium homeostasis, and protection against oxidative stress [114]. It is worth highlighting another study, not included in the present systematic review, published in Italian, which verified elevated calgranulin A and C saliva concentrations in patients with FMS [115].

Research indicates that the consumption of extra-virgin olive oil (EVOO) acts protectively in balancing redox homeostasis and tampering down inflammation [116, 117]. This is mostly due to the phenolic content of EVOO, and in particular hydroxy-tyrosol (HT). Interestingly, a preliminary study [118] assessed the effect of a high-HT nutritional treatment to the proteome of dermal fibroblasts of a single patient with FMS, versus a healthy control. The results revealed that treatment with HT normalized the differential expression in proteins involved in the turnover of extracellular matrix and oxidative metabolism, observed in the patient with FMS, against the healthy control [118]. Although greatly underpowered, this study highlighted a possible therapeutic pathway for FMS, in need of more high-quality research. Other research groups have also identified EVOO as an important adjuvant in FMS treatment. In a RCT [119], women with FMS treated with 50 mL of EVOO or other ROO for a period of 3 weeks showed improvements in their antioxidative profiles (protein carbonyls, lipid peroxidation) and pain (FIQ), and QoL scales, exhibiting antithrombotic and anti-inflammatory properties [103].

### Immune Response Biomarkers

Serum amyloid (P component) was elevated in plasma and serum, while serum amyloid (SA4) was high in plasma and low in serum. These proteins belong to the acute phase proteins and regulate the immune response. Different saliva and plasma concentrations of Ig kappa chain C region and Ig alpha-1 chain C region immunoglobulin segments were observed in patients with FMS. In parallel, Ig lambda-2 chain C region segments were high in saliva and low in serum samples. To date, the exact role of the immune system in pain development and sensation remains unclear [120]. Nonetheless, an increased incidence of immunodeficiency has been observed in patients with FMS, and conversely, FMS tends to be more common in patients with primary immunodeficiency [121, 122]. Furthermore, immune aberrations have been reported in FMS, which were considered partially responsible for the sensation of pain [123•].

PGAM1 is an enzyme of glycolysis, and serum autoantibodies against PGAM1 have been reported in autoimmune hepatitis and various neurological diseases, including multiple sclerosis and neuromyelitis optica [55, 124]. As seen in the present results, PGAM1 concentrations were also increased in the saliva of patients with FMS. As the salivary gland innervates directly from the trigeminovascular system, saliva contains several neuropeptides, potentially providing information regarding CNS pathology and related disorders [125, 126]. In this manner, the elevated PGAM1 saliva levels could be of particular interest, as FMS has been associated with several neurological disorders [127]. More research is required to delineate this association.

### Cardiovascular Risk Biomarkers

Profilin-1 is an actin-binding protein that regulates DNA damage response and repair mechanisms [73]. Saliva samples of patients with FMS exhibited higher profilin-1 concentrations than controls, whereas lower profilin-1 concentrations were observed in serum samples. In parallel, it has been suggested [45•] that profilin-1 could identify patients with FMS, depending on the main reported symptom (pain or stiffness). Recently, profilin-1 emerged as a new player in the field of atherosclerosis; it is accumulated in high concentrations in stable atherosclerotic plaques and thrombi from infarct-related arteries in cases of acute myocardial infarction [128]. Furthermore, several studies have reported histological abnormalities in the muscle tissue of patients with FMS, indicative of microvascular dysfunction, including capillary dysfunction and myocyte mitochondric abnormalities [106, 129–131].

Finally, different apolipoprotein C-III concentrations were reported in the plasma and CSF of patients with FMS. Apolipoprotein C-III is an atherogenic protein that leads to higher triglyceride levels, resulting in augmented risk of cardiovascular events. Its role in the CNS remains unclear. Higher serum total cholesterol, LDL, and triglyceride levels have been observed in women with FMS, and these patients are known to have increased cardiovascular risk in parallel to obesity [132–134].

### Other Biomarkers with Multiple Roles

A common protease inhibitor involved in the coagulation cascade, inflammation, and autoimmunity phenomena is α2-macroglobulin [135]. Plasma levels of α2-macroglobulin were elevated in patients with FMS compared with healthy controls. A2-macroglobulin has been detected in the proteome of patients with chronic fatigue syndrome [136] and multiple sclerosis [137]. Interestingly, new research and advances in pain management have suggested using α2-macroglobulin for treating neuropathic pain [138•, 139]. As a result, α2-macroglobulin injections are frequently applied for managing knee osteoarthritis [140], as they prevent cartilage degeneration by inhibiting catabolic enzymes and cytokines [141].

### Associations Between Proteomics and QoL of Patients

The present systematic review aimed to identify potential relationships between specific proteomic markers in FMS and QoL scales; however, for the most part, the results were inconclusive. One study using plasma samples [44] associated elevated α2-macroglobulin and TRFE with moderate and severe pain intensity. Finally, another study using serum samples [45•] concluded that different clinical profiles may be associated with different proteomic markers.

### Combining Biomarkers for Diagnosis and Treatment

The quest for diagnostic biomarkers for FMS continues, aiming to provide a critical step towards prompt intervention [96]. Annemans [22] revealed that establishing an FMS diagnosis decreases the financial costs associated with tests and imaging, referrals, doctor visits, and pharmaceuticals, showing that a diagnosis reduces the use of resources. Thus, efforts to identify and combine diagnostic markers are warranted to reduce the burden and costs associated with FMS. It is important to note, that although several different proteins were identified herein, it is difficult to understand if the same biological pathways act as triggers for the development of FMS, or if different pathways result in the same disease phenotype for different patients. Thus, we are currently unsure which combination of biomarkers can offer improved diagnostic ability for FMS, or whether a combination therapy (antioxidants, iron, antithrombotic, etc.) conferring a comprehensive synergistic action can tamper down FMS symptoms. What is known though from an early study [142], is that approximately 30% of patients with FMS have reported taking dietary supplements or making holistic dietary changes in response to their disease, according to their healthcare professionals, all achieving improved pain relief. As for the diagnosis, one of the studies [46] included herein reported using a decision tree model to differentiate patients with FMS and controls based on the expression levels of histidine protein methyltransferase 1 homolog (HMPT1), Interleukin-1 receptor accessory protein (IL1RAP) and Ig lambda chain V-IV region (IGL3-25), yielding an accuracy of up to 0.97. No other efforts to achieve improved diagnostic accuracy were reported by the authors of the remaining proteomics studies, and as quantitative data were not included in all research, we were unable to conduct this either.

### Limitations of the Included Studies

Some of the included studies shared common authors. Specifically, the two studies using CSF samples [41, 42] had the same first author and two additional authors in common. Also, the two studies investigating proteomics in saliva [38, 39] were conducted by the same research group core, sharing five common authors, with one study essentially being the extension of the other, as it included a healthy population and a group of patients with chronic pain, as controls. This could be considered a confounding factor.

Most research items failed to report the medications taken by the patients or controls. Drugs can affect the proteome, as they have various actions on the metabolism and excretion of proteins from the body.

Finally, there were great differences in the proteomics methodologies among the studies. Distinct methods have varying sensitivity and specificity and may produce different results in the proteins detected and quantified. Each method has its advantages and limitations and may excel in different areas of proteomic research. Finally, the inherent differences in sample preparation, separation, and detection can lead to variations in the total number of proteins detected.

## Conclusions

In summary, proteomics consist of a useful tool, providing insight into the processes and signaling pathways that may be involved in the pathogenesis of FMS. In the present systematic review, the proteome of patients with FMS was studied, and specific protein expression patterns were identified. Proteins related to the complement cascade, the coagulation cascade, inflammation, the immune system, iron metabolism, and the oxidative stress process were found to be dysregulated in FMS patients. FMS appears closely related to the oxidative stress pathway, as many proteins protecting the body from oxidative stress appear are dysregulated. However, more primary studies are required to aid our understanding of this association.

### Supplementary Information

Below is the link to the electronic supplementary material.

Supplementary file1 (PDF 224 KB)

## Data Availability

Extracted data for this review are available upon request to the corresponding author.
