# Supplementary material for: Proteomics in Patients with Fibromyalgia Syndrome: A Systematic Review of Observational Studies
Source: Curr Pain Headache Rep. 2024 Apr 23;28(7):565–86. doi: 10.1007/s11916-024-01244-4 (PMC11271354; doi:10.1007/s11916-024-01244-4)
Supplement: Supplementary file 1 — Supplementary file1 (PDF 224 KB) [file 11916_2024_1244_MOESM1_ESM.pdf]

Supplementary tables

Supplementary Table 1. PEO components of the study's research question.

P	Population	Adult patients
E	Exposure	Fibromyalgia
O	Outcomes	- Identification of proteins isolated in plasma, serum, saliva, cerebrospinal fluid with proteomics methodologies<br>- Association of such proteins with age, gender, disease activity scores, quality of life scores and severity of symptoms

PEO, Population – Exposure – Outcomes.

Supplementary Table 2. Basic terms and key words used in the search strategy.

Terms	Fibromyalgia	Proteomics	Biologic fluids
**Free text**	Fibromyalgia [tiab]	Proteomic* [tiab]	Serum [tiab]
	FM [tiab]	Proteome* [tiab]	Saliva* [tiab]
	FMS [tiab]	Biomarker* [tiab]	Plasma [tiab]
	Fibromyositis [tiab]	Peptide [tiab]	Cerebrospinal fluid [tiab]
	Fibrositis [tiab]		CSF [tiab]
			Blood [tiab]
**MeSH Terms**	Fibromyalgia"[MeSH Terms]	Proteomics"[MeSH Terms]	
**Excluded**	NOT animal		

CSF, Cerebrospinal fluid; FM, fibromyalgia; FMS, fibromyalgia syndrome; MeSH, Medical Subject Headings.

Supplementary Table 3. Search syntaxes used in PubMed and CENTRAL databases.

PubMed	((((((((Fibromyalgia[Title/Abstract]) OR (FM[Title/Abstract])) OR (FMS[Title/Abstract])) OR (Fibrositis[Title/Abstract])) OR (fibromyositis[Title/Abstract])) OR (fibromyalgia[MeSH Terms])) AND (((((proteomics[MeSH Terms]) OR (proteomic*[Title/Abstract])) OR (proteome*[Title/Abstract])) OR (biomarker*[Title/Abstract])) OR (peptide[Title/Abstract]))) AND ((((((serum[Title/Abstract]) OR (saliva*[Title/Abstract])) OR (CSF[Title/Abstract])) OR (cerebrospinal fluid[Title/Abstract])) OR (plasma[Title/Abstract])) OR (blood[Title/Abstract]))) NOT (animal[Title/Abstract])
CENTRAL	#1 (fibromyalgia):ti,ab,kw =3407<br>#2(FMS):ti,ab,kw =832<br>#3(Fibromyositis):ti,ab,kw =4<br>#4(fibrositis):ti,ab,kw =981<br>#5 MeSH descriptor: [Fibromyalgia] explode all trees =1610<br>#6 #1 OR #2 OR #3 OR #4 OR #5 =4958<br>#7 (proteomic*):ti,ab,kw =1263<br>#8 (proteome):ti,ab,kw =1422<br>#9 (biomarker*):ti,ab,kw =47531<br>#10 MeSH descriptor [Proteomics] in all MeSH products =165<br>#11 #7 OR #8 OR #9 OR #10 =48414<br>#12 (blood):ti,ab,kw =405438<br>#13 (plasma):ti,ab,kw 108769<br>#14 (CSF):ti,ab,kw =8795<br>#15 (Cerebrospinal fluid):ti,ab,kw =4545<br>#16 (saliva*):ti,ab,kw = 13290<br>#17 (serum):ti,ab,kw =114885<br>#18 #12 OR #13 OR #14 OR #15 OR #16 OR #17 =490316<br>#19 #6 AND #11 AND #18 =131

CENTRAL, Cochrane Central Register of Controlled Trials.

Supplementary Table 4. List of excluded studies.

First author, publication year	Title	Reason for exclusion
Al-Nimer M, 2018	Serum levels of serotonin as a biomarker of newly diagnosed fibromyalgia in women: Its relation to the platelet indices.	Not proteomics, uses ELISA to measure serotonin
Anderberg U, 1999	Elevated plasma levels of neuropeptide Y in female fibromyalgia patients.	No use of proteomics, RIA
Ang D, 2011	MCP-1 and IL-8 as pain biomarkers in fibromyalgia: a pilot study.	No use of proteomics
Applbaum E, 2019	Novel Sjögren's autoantibodies found in fibromyalgia patients with sicca and/or xerostomia.	No use of proteomics
Bazzichi L, 2013	A multidisciplinary approach to study the effects of balneotherapy and mud-bath therapy treatments on fibromyalgia.	RCT, non-observational
Bennett R, 1990	Low levels of somatomedin C in patients with the fibromyalgia syndrome. A possible link between sleep and muscle pain.	No use of proteomics, RIA
Bjersing J, 2012	Changes in pain and insulin-like growth factor 1 in fibromyalgia during exercise: the involvement of cerebrospinal inflammatory factors and neuropeptides.	RCT, non-observational, non- proteomics, ELISA
Bjersing J, 2017	Benefits of resistance exercise in lean women with fibromyalgia: involvement of IGF-1 and leptin.	Substudy of an RCT, of interventional design, no proteomics results, ELISA
Blanco I, 2010	Low plasma levels of monocyte chemoattractant protein-1 (MCP-1), tumor necrosis factor-alpha (TNFalpha), and vascular endothelial growth factor (VEGF) in patients with alpha1-antitrypsin deficiency-related fibromyalgia.	No use of proteomics, assesses MCP1, TNFa and VEGF in AAT deficient FM and HC with ELISA
Bote M, 2012	Inflammatory/stress feedback dysregulation in women with fibromyalgia.	No use of proteomics
Bozkurt M, 2014	Serum prolidase enzyme activity and oxidative status in patients with fibromyalgia.	No use of proteomics
Caboni P, 2014	Metabolomics analysis and modeling suggest a lysophosphocholines-PAF receptor interaction in fibromyalgia.	Lipidomics
Cê P, 2018	Salivary Levels of Interleukin-1β in Temporomandibular Disorders and Fibromyalgia.	Uses ELISA for IL-1b, no use of proteomics
Clos-Garcia M, 2019	Gut microbiome and serum metabolome analyses identify molecular biomarkers and altered glutamate metabolism in fibromyalgia.	Not relevant results
Cordero M, 2014	NLRP3 inflammasome is activated in fibromyalgia: the effect of coenzyme Q10.	No use of proteomics

First author, publication year	Title	Reason for exclusion
Costa A, 2022	Heart Rate Variability and Salivary Biomarkers Differences between Fibromyalgia and Healthy Participants after an Exercise Fatigue Protocol: An Experimental Study.	No use of proteomics
Čulić O, 2016	Serum activities of adenosine deaminase, dipeptidyl peptidase IV and prolyl endopeptidase in patients with fibromyalgia: diagnostic implications.	No use of proteomics
Dos Santos J, 2022	Oxidative Stress Biomarkers and Quality of Life Are Contributing Factors of Muscle Pain and Lean Body Mass in Patients with Fibromyalgia.	No use of proteomics
Elkfury J, 2021	Dysfunctional eating behavior in fibromyalgia and its association with serum biomarkers of brain plasticity (BDNF and S100B): an exploratory study.	No use of proteomics
Furer V, 2018	Elevated Levels of Eotaxin-2 in Serum of Fibromyalgia Patients.	No use of proteomics, assesses specific protein levels with ELISA
Garcia J, 2014	Soluble fractalkine in the plasma of fibromyalgia patients.	ELISA, not proteomics
Garcia J, 2014	Altered profile of chemokines in fibromyalgia patients.	ELISA, not proteomics
Gerra M, 2021	A family-based study to identify genetic biomarkers of fibromyalgia: consideration of patients' subgroups.	Gene analysis, no use of proteomics
Giacomelli C, 2011	[MALDI-TOF and SELDI-TOF analysis: "tandem" techniques to identify potential biomarker in fibromyalgia].	Foreign language
Hackshaw K, 2019	Metabolic fingerprinting for diagnosis of fibromyalgia and other rheumatologic disorders.	Not relevant results
Höcherl K, 2000	Effect of tropisetron on circulating catecholamines and other putative biochemical markers in serum of patients with fibromyalgia.	No use of proteomics, ELISA
Iannuccelli C, 2010	Pathophysiology of fibromyalgia: a comparison with the tension-type headache, a localized pain syndrome.	Assesses cytokines in TTH, FM, HC. No proteomics
Iannuccelli C, 2022	Gender influence on clinical manifestations, depressive symptoms and brain-derived neurotrophic factor (BDNF) serum levels in patients affected by fibromyalgia.	No use of proteomics, measures BDNF with ELISA and compares between FM/HC and F/M
Jacobsen S, 1990	Primary fibromyalgia: clinical parameters in relation to serum procollagen type III aminoterminal peptide.	No use of proteomics
Jensen L, 1988	Serum procollagen type III aminoterminal peptide in primary fibromyalgia (fibrositis syndrome).	No use of proteomics, RIA

First author, publication year	Title	Reason for exclusion
Karatas G, 2020	Dynamic thiol and disulphide homoeostasis in fibromyalgia.	No use of proteomics, assesses the imbalance between thiol/disulfide levels between FM and a controls
Khamisy-Farah R, 2021	Inflammatory Markers in the Diagnosis of Fibromyalgia.	No use of proteomics
Koca T, 2019	The Importance of G-protein Coupled Estrogen Receptor in Patients With Fibromyalgia.	No use of proteomics
Korucu R, 2020	Serum Calcitonin Gene-Related Peptide and Receptor Protein Levels in Patients With Fibromyalgia Syndrome: A Cross-Sectional Study.	No use of proteomics
Kutu F, 2019	Pro-inflammatory Cytokines and Oxidized Low-Density-Lipoprotein in Patients With Fibromyalgia.	No use of proteomics, measures specific protein levels with ELISA
Lin X, 2022	AKAP12 and RNF11 as Diagnostic Markers of Fibromyalgia and Their Correlation with Immune Infiltration.	Uses artificial intelligence to test genes that could be related with FM. No use of proteomics.
Martínez-Lara A, 2020	Mitochondrial Imbalance as a New Approach to the Study of Fibromyalgia.	No use of proteomics
Mease P, 2009	Fibromyalgia syndrome module at OMERACT 9: domain construct.	No use of proteomics
Menzies V, 2013	Psychoneuroimmunological relationships in women with fibromyalgia.	No proteomics, correlates blood parameters assessed with ELISA with questionnaires and symptoms
NCT00810329	Proteomics of Cerebrospinal Fluid in Chronic Fatigue Syndrome.	Pooling FM patients with CFS
Otero M, 2005	Ghrelin plasmatic levels in patients with fibromyalgia.	No use of proteomics, RIA
Raffaeli W, 2020	Identification of MOR-Positive B Cell as Possible Innovative Biomarker (Mu Lympho-Marker) for Chronic Pain Diagnosis in Patients with Fibromyalgia and Osteoarthritis Diseases.	Flow cytometry, not proteomics
Ranzolin A, 2016	Evaluation of cytokines, oxidative stress markers and brain-derived neurotrophic factor in patients with fibromyalgia - A controlled cross-sectional study.	No use of proteomics, ELISA
Ribeiro V, 2018	Inflammatory biomarkers responses after acute whole body vibration in fibromyalgia.	No use of proteomics, ELISA

First author, publication year	Title	Reason for exclusion
Rus A, 2016	Nitric Oxide, Inflammation, Lipid Profile, and Cortisol in Normal- and Overweight Women with Fibromyalgia.	No use of proteomics
Rus A, 2022	CGRP, VEGF, and Clinical Manifestations in Women with Fibromyalgia.	No use of proteomics, measures specific protein levels with ELISA
Sarchielli, P, 2006	Glial Cell Line-Derived Neurotrophic Factor and Somatostatin Levels in Cerebrospinal Fluid of Patients Affected by Chronic Migraine and Fibromyalgia.	No use of proteomics
Soldatelli M, 2021	Mapping of predictors of the disengagement of the descending inhibitory pain modulation system in fibromyalgia: an exploratory study.	No use of proteomics
Stensson, 2018	The Relationship of Endocannabinoidome Lipid Mediators with Pain and Psychological Stress in Women with Fibromyalgia: A Case-Control Study.	Lipidomics
Sugimoto C, 2015	Mucosal-associated invariant T cell is a potential marker to distinguish fibromyalgia syndrome from arthritis.	No use of proteomics, flow cytometry
Tander B, 2007	Serum ghrelin levels but not GH, IGF-1 and IGFBP-3 levels are altered in patients with fibromyalgia syndrome.	Uses ELISA and RIA, no use of proteomics
Theoharides T, 2009	Mast Cells, Neuroinflammation and Pain in Fibromyalgia Syndrome.	Literature review, no use of proteomics methodology
Vaerøy H, 1989	Modulation of pain in fibromyalgia (fibrositis syndrome): cerebrospinal fluid (CSF) investigation of pain related neuropeptides with special reference to calcitonin gene related peptide (CGRP).	No use of proteomics, RIA

AAT, alpha1-antitrypsin; AKAP12, A-Kinase Anchoring Protein 12; BDNF, brain-derived neurotrophic factor; CGRP, calcitonin gene related peptide; CFS, Chronic Fatigue Syndrome; CSF, Cerebrospinal fluid; ELISA, enzyme-linked immunoassay; FM, fibromyalgia; GH, growth hormone; HC, healthy controls; IGF-1, insulin-growth factor 1; IGFBP-3, Insulin-Like Growth Factor-Binding Protein 3; IL-1β, Interleukin-1β; IL-8, interleukin 8; MCP-1, monocyte chemoattractant protein-1; OMERACT, outcome measures in rheumatology; PAF, Platelet-activating factor; RCT, randomized-controlled trial; RIA, radioimmunoassay; RNF-11, RING Finger 11; TNFα, tumor necrosis factor-alpha; TTH, tension-type headache; VEGF, vascular endothelial growth factor.

Supplementary Table 5. Exclusion criteria used for the selection of participants with FMS in each study.

Study	Exclusion criteria
Bazzichi [39]	Other connective tissue diseases, severe heart, lung, liver or kidney diseases, secondary FMS.
Ciregia [38]	Patients older than 65 years, potential or existing pregnancy and lactation, active rheumatic disease, psychiatric disorders, infectious diseases.
Fineschi [37]	Chronic inflammatory diseases, autoimmune diseases, malignancy, major depression, use of corticosteroids or immunosuppressants, active infection, positive ANA, high CRP, positive IgM, RF and anti-CCP.
Han [46]	Psychiatric disorders (schizophrenia, schizotypal disorder), alcohol or substance abuse, active malignancy, major rheumatic disease (SLE, RA, SS, SpA), pregnancy.
Hsu [45]	Active infection, malignancy or history of major surgery.
Khoonsari [41]	Psychiatric illnesses, dementia, epilepsy, alcohol or substance abuse.
Khoonsari [42]	Patients with RA had no neurological diseases, while the other group had no chronic pain disorders.
Ramirez-Tejero [40]	DM, hypertension, cancer, ischemic heart disease, pregnancy, lactation, obesity.
Ruggiero [43]	Severe comorbidities.
Wahlen [44]	Hypertension, osteoarthritis of the hip or knee, alcohol abuse, participation in a rehabilitation program within the previous year, regular exercise (resistance exercise), inability to understand the language, NSAIDs or hypnotics, rheumatic and neurological diseases (RA, SLE, MS), DM, CVD, serious psychiatric disorders (psychosis, major depressive disorder, anxiety disorders).

ANA, antinuclear antibodies; anti-CCP, Anti-cyclic citrullinated peptide; BDNF, brain-derived neurotrophic factor; CRP, c-reactive protein; CVD, cardiovascular disease; DM, diabetes mellitus; FMS, fibromyalgia syndrome; IgM, Immunoglobulin M; MS, multiple sclerosis; NSAIDs, Non-steroidal anti-inflammatory drugs; RA, rheumatoid arthritis; RF, rheumatoid factor; SLE, systematic lupus erythematosus; SpA, ankylosing spondylitis; SS, Sjögren's syndrome.

Supplementary Table 6. Significance (*p*-values) and fold change in proteins observed in saliva samples of patients with FMS against other groups.

Protein name	Abbreviation	UniProt	vs. healthy controls		vs. Rheumatoid arthritis		vs. Migraine		Reference
			p-value	Fold change	*p*-value	Fold change	*p*-value	Fold change	
Transaldolase	TALDO	P37837	<0.0001	+3.02	-	-	-	-	Bazzichi [39]
Phosphoglycerate mutase-1	PGAM1	P18669	0.0011	+2.5	-	-	-	-	Bazzichi [39]
Proteasome subunit-α-type-2	PSMA2	P25787	0.026	+3.4	-	-	-	-	Bazzichi [39]
Rho GDP-dissociation inhibitor 2	RhoGDI2	P52566	0.024	+2	-	-	-	-	Bazzichi [39]
Haptoglobin-related protein precursor	HPR	P00739	0.003	+5.2	-	-	-	-	Bazzichi [39]
Cofilin-1	CFL1	P23528	0.07	+2.7	-	-	-	-	Bazzichi [39]
Cyclophilin A	CyPA	P62937	0.05	+2.1	-	-	-	-	Bazzichi [39]
Cyclophilin A	CyPA	P62937	0.016	+2.1	-	-	-	-	Bazzichi [39]
Profilin-1	PFN1	P07737	0.018	+2	-	-	-	-	Bazzichi [39]
Calgranulin A	S100-A8	P05109	0.0036	+2.6	-	-	-	-	Bazzichi [39]
Calgranulin A	S100-A8	P05109	0.0002	+4.7	-	-	-	-	Bazzichi [39]
Calgranulin C	S100-A12	P80511	0.001	+12.9	-	-	-	-	Bazzichi [39]
Gelsolin	*GSN*	*A2A418*	*NS*	*+1.2*	-	-	-	-	*Bazzichi* [39]
α-amylase 1	AMY1A	P04745	0.0012	+1.6	NS	NS	0.0045	+1.6	Ciregia [38]
α-amylase 1	AMY1A	P04745	0.0002	+1.8	0.006	-1.5	0.0024	+1.4	Ciregia [38]
α-amylase 1	AMY1A	P04745	0.00025	+2.3	0.04	-1.3	0.026	+1.6	Ciregia [38]
α-amylase 1	AMY1A	P04745	0.0035	+2.5	0.0025	-2.1	NS	NS	Ciregia [38]
α-amylase 1	AMY1A	P04745	0.002	+2.0	0.0054	-1.9	0.045	+1.6	Ciregia [38]
α-amylase 1	AMY1A	P04745	0.00027	+2.4	0.013	-1.7	0.009	+1.8	Ciregia [38]
α-enolase	ENO1	P06733	0.0013	+1.7	0.03	+1.3	0.009	+1.6	Ciregia [38]
Glucosamine-6-phosphate isomerase 1	*GNP1*	*P46926*	*NS*	*NS*	*0.0011*	*-2*	*0.012*	*-1.6*	*Ciregia* [38]

Protein name	Abbreviation	UniProt	vs. healthy controls		vs. Rheumatoid arthritis		vs. Migraine		Reference
			p-value	Fold change	*p*-value	Fold change	*p*-value	Fold change	
Glutathione S-transferase P	GSTP	P09211	0.005	+1.7	NS	NS	NS	NS	Ciregia [38]
Heat shock protein β-1	*HSPB1*	*P04792*	*NS*	*NS*	*0.009*	*-1.7*	*NS*	*NS*	*Ciregia* [38]
Ig α-1 chain C region	*IgAc1*	*P01876*	*NS*	*NS*	*0.000032*	*-3.2*	*NS*	*NS*	*Ciregia* [38]
Ig α-1 chain C region	*IgAc1*	*P01876*	*NS*	*NS*	*0.000037*	*-2*	*0.015*	*+1.4*	*Ciregia* [38]
Ig κ chain C region	IgKC	P01834	0.001	+1.5	NS	NS	0.0007	+1.6	Ciregia [38]
Ig λ-2 chain C regions	Iglc2	P0CG05	0.0012	+1.5	NS	NS	0.016	+1.4	Ciregia [38]
Phosphoglycerate mutase 1	PGAM1	P18669	0.032	+1.3	NS	NS	0.04	+1.3	Ciregia [38]
Transaldolase	TALDO	P37837	0.026	+1.4	NS	NS	NS	NS	Ciregia [38]
Polymeric Ig receptor precursor	pIgR	P01833	0.012	-1.5	0.007	+2	NS	NS	Ciregia [38]
Polymeric Ig receptor precursor	pIgR	P01833	0.014	-1.4	NS	NS	NS	NS	Ciregia [38]
Polymeric Ig receptor precursor	pIgR	P01833	0.003	-1.6	0.007	+1.8	NS	NS	Ciregia [38]
Serotransferrin	TRFE	P02787	0.000007	+2	0.004	+2.1	1.00e-06	+2.4	Ciregia [38]
Serotransferrin	TRFE	P02787	0.003	+2	0.004	+2.1	1.70e-06	+2.7	Ciregia [38]
Serotransferrin	TRFE	P02787	0.009	+1.6	0.00019	+2.3	1.30e-06	+2	Ciregia [38]
Serotransferrin	TRFE	P02787	0.00012	+1.7	0.003	+2.1	9.00e-06	+2.1	Ciregia [38]
Serum albumin	ALB	P02768	0.0085	+2.4	NS	NS	0.008	+2.4	Ciregia [38]
Serum albumin	ALB	P02768	0.00033	+2.3	0.0014	-2.1	0.039	+1.6	Ciregia [38]
Cystatin-SN	CYS	P01037	0.001	-2	NS	NS	2.0e-05	-2.1	Ciregia [38]
Cystatin-SN	*CYS*	*P01037*	*NS*	*NS*	*NS*	*NS*	*0.02*	*-1.5*	*Ciregia* [38]
Calgranulin-A	S100-A8	P05109	0.03	+1.4	NS	NS	NS	NS	Ciregia [38]

FMS, fibromyalgia syndrome; GDP, guanosine diphosphate; Ig, immunoglobulin; UniProt, Universal Protein Resource.

Proteins appearing more than one times correspond to different fragments. Proteins in blue and italic fonts were not significant compared to healthy controls.

Supplementary Table 7. Fold change, Q-value and VIP in proteins found in CSF samples of patients with FMS compared to controls.

Protein name	Abbreviation	UniProt	Q-value	Fold change*	VIP	Reference
Apolipoprotein C-III	ApoC-III	P02656	2.16E-3	+2.3	-	Khoonsari [41]
Galectin-3-binding protein	LG3BP	Q08380	3.07E-2	+1.05	-	Khoonsari [41]
ProSAAS	PCKS1	P29120	2.16E-2	+0.75	-	Khoonsari [41]
Malate dehydrogenase	MDH	P40925	2.11E-5	-2.03	-	Khoonsari [41]
Neural cell adhesion molecule L1	L1CAM	P32004	-	-	1.9	Khoonsari [42]
Complement C4-A	C4A	P0C0L4	-	-	1.8	Khoonsari [42]
Lysozyme C	LYSC	P61626	-	-	1.7	Khoonsari [42]
Receptor-type tyrosine-protein phosphatase zeta	PTPRz	P23471	-	-	1.7	Khoonsari [42]
Apolipoprotein D	ApoD	P05090	-	-	1.6	Khoonsari [42]
α-1-antichymotrypsin	α1ACT	P01011	-	-	1.5	Khoonsari [42]
Progranulin	PGRN	P28799	-	-	1.5	Khoonsari [42]
Pro-low-density lipoprotein receptor-related protein 1	LRP1	Q07954	-	-	1.4	Khoonsari [42]
Calcium/calmodulin-dependent protein kinase type II subunit α	CaMKIIα	Unknown	-	-	1.5	Khoonsari [42]
c-Kit	c-Kit	A0A0U2N547	-	-	1.5	Khoonsari [42]

*, fold change presented in logarithmic scale (log2). CSF; Cerebrospinal fluid; FMS, fibromyalgia syndrome; UniProt, Universal Protein Resource; VIP, variable importance in projection.

Proteins with a VIP > 1.3 were considered as the most discriminative ones.

Supplementary Table 8. Significance (*p*-values), pcorr, fold change and VIP in proteins observed in plasma samples of patients with FMS against controls.

Protein name	Abbreviation	UniProt	*p*-value	pcorr*	Fold change	VIP**	Reference
Haptoglobin	HP	P00738	7.95E-05	-	2.54	-	Ramirez-Tejero [40]
Fibrinogen γ chain	FGG	P02679	5.49E-03	-	2.3	-	Ramirez-Tejero [40]
Actin, cytoplasmic 1	ACT	P60709	1.11E-03	-	2.26	-	Ramirez-Tejero [40]
Thrombospondin-1	THBS1	P07996	3.33E-02	-	2.21	-	Ramirez-Tejero [40]
Apolipoprotein L1	APOL1	O14791	3.77E-04	-	1.91	-	Ramirez-Tejero [40]
Serum amyloid A-4 protein	SAA4	P35542	3.43E-03	-	1.89	-	Ramirez-Tejero [40]
Apolipoprotein C-II	APOC2	P02655	1.15E-02	-	1.83	-	Ramirez-Tejero [40]
Serotransferrin	TRFE	P02787	1.30E-03	-	1.83	-	Ramirez-Tejero [40]
Fibrinogen β chain	FGB	P02675	4.38E-02	-	1.78	-	Ramirez-Tejero [40]
Pregnancy zone protein	PZP	P20742	4.05E-03	-	1.76	-	Ramirez-Tejero [40]
α-1-acid glycoprotein 2	A1AGP2	P19652	4.58E-03	-	1.74	-	Ramirez-Tejero [40]
Ig α-1 chain C region	IGAC	P01876	3.09E-02	-	1.74	-	Ramirez-Tejero [40]
Coagulation factor X	X	P00742	9.92E-03	-	1.63	-	Ramirez-Tejero [40]
α-1-acid glycoprotein 1	A1AGP1	P02763	2.27E-02	-	1.58	-	Ramirez-Tejero [40]
α-2-macroglobulin	A2M	P01023	1.20E-02	-	1.52		Ramirez-Tejero [40]
Ig mu chain C region	IGHM	P01871	2.24E-02	-	1.52	-	Ramirez-Tejero [40]
Complement C1s subcomponent	C1S	P09871	1.23E-04	-	1.49	-	Ramirez-Tejero [40]
Serum amyloid P-component	SAP	P02743	2.44E-02	-	1.48	-	Ramirez-Tejero [40]
Complement factor H	CFAH	P08603	4.20E-03	-	1.43	-	Ramirez-Tejero [40]
Complement component C7	C7	P10643	9.26E-03	-	1.39	-	Ramirez-Tejero [40]
Complement C2	C2	P06681	9.72E-03	-	1.38	-	Ramirez-Tejero [40]
Vitamin K-dependent protein S	PROS	P07225	1.06E-02	-	1.38	-	Ramirez-Tejero [40]
Coagulation factor IX	IX	P00740	7.09E-03	-	1.36	-	Ramirez-Tejero [40]
Complement C1q subcomponent subunit C	C1Qc	P02747	1.55E-02	-	1.35	-	Ramirez-Tejero [40]

Protein name	Abbreviation	UniProt	*p*-value	pcorr*	Fold change	VIP**	Reference
Complement component C9	C9	P02748	7.31E-03	-	1.33	-	Ramirez-Tejero [40]
Angiotensinogen	AGT	P01019	3.98E-02	-	0.73	-	Ramirez-Tejero [40]
Histidine-rich glycoprotein	HRG	P04196	2.90E-02	-	0.7	-	Ramirez-Tejero [40]
Monocyte differentiation antigen CD14	CD14	P08571	5.95E-03	-	0.67	-	Ramirez-Tejero [40]
Corticosteroid-binding globulin	CBG	P08185	2.78E-02	-	0.66	-	Ramirez-Tejero [40]
Thyroxine-binding globulin	THBG	P05543	5.93E-03	-	0.63	-	Ramirez-Tejero [40]
Protein Z-dependent protease inhibitor	ZPI	Q9UK55	1.29E-02	-	0.63	-	Ramirez-Tejero [40]
Fetuin-B	FETUB	Q9UGM5	3.75E-03	-	0.53	-	Ramirez-Tejero [40]
Carbonic anhydrase 1	CA	P00915	4.06E-03	-	0.47	-	Ramirez-Tejero [40]
Ig κ chain C region	IgKC	P01834	-	-0.56	0.40	1.92	Wahlen [44]
Serotransferrin	TRFE	P02787	-	+0.47	1.50	1.61	Wahlen [44]
α-2-antiplasmin	A2AP	P08697	-	+0.46	2.04	1.58	Wahlen [44]
Complement C4-B (fragment)	B4B	P0C0L5	-	-0.45	0.71	1.57	Wahlen [44]
Fibrinogen α chain (fragment)	FGA	P02671	-	-0.45	0.27	1.57	Wahlen [44]
Haptoglobin	HP	P00738	-	+0.44	1.26	1.51	Wahlen [44]
Fibrinogen α chain	FGA	P02671	-	-0.43	0.34	1.49	Wahlen [44]
Serotransferrin	TRFE	P02787	-	+0.43	1.17	1.48	Wahlen [44]
Complement C4-B (fragment)	C4B	P0C0L5	-	+0.43	1.27	1.48	Wahlen [44]
Fibrinogen β chain	FGB	P02675	-	+0.42	1.59	1.45	Wahlen [44]
α-1B-glycoprotein	A1BG	P04217	-	-0.41	0.87	1.43	Wahlen [44]
Fibrinogen α chain (fragment)	FGA	P02671	-	-0.4	0.55	1.37	Wahlen [44]
Plasminogen	PLG	P00747	-	-0.4	0.84	1.37	Wahlen [44]
Complement C3 β chain	C3B	P01024	-	+0.39	1.19	1.33	Wahlen [44]
Kininogen-1	KNG1	P01042	-	-0.38	0.72	1.33	Wahlen [44]
Plasminogen	PLG	P00747	-	-0.38	0.94	1.3	Wahlen [44]

Protein name	Abbreviation	UniProt	*p*-value	pcorr*	Fold change	VIP**	Reference
α-2-antiplasmin	A2AP	P08697	-	-0.37	0.66	1.28	Wahlen [44]
α-2-macroglobulin	A2M	P01023	-	-0.37	0.41	1.28	Wahlen [44]
α-2-antiplasmin	A2AP	P08697	-	-0.36	0.84	1.24	Wahlen [44]
Fibrinogen α chain	FGA	P02671	-	-0.36	0.44	1.24	Wahlen [44]
Complement C1r subcomponent	C1R	P00736	-	-0.34	0.85	1.17	Wahlen [44]
Haptoglobin	HP	P00738	-	+0.33	1.24	1.14	Wahlen [44]
Plasminogen	PLG	P00747	-	-0.33	0.91	1.12	Wahlen [44]
Plasminogen	PLG	P00747	-	-0.32	0.74	1.11	Wahlen [44]
Ig κ chain C region	IgKC	P01834	-	-0.32	0.62	1.11	Wahlen [44]
β-2-glycoprotein 1	B2GP	P02749	-	+0.31	1.10	1.06	Wahlen [44]
Fibrinogen α chain (fragment)	FGA	P02671	-	-0.3	0.26	1.05	Wahlen [44]
α-2-macroglobulin	A2M	P01023	-	-0.3	0.65	1.05	Wahlen [44]
Complement factor I light chain		P05156	-	-0.3	0.88	1.05	Wahlen [44]
Gelsolin	GSN	P06396	-	+0.3	1.01	1.03	Wahlen [44]
Apolipoprotein C-III	APOC3	P02656	-	-0.29	0.33	1.01	Wahlen [44]
Complement C3b	C3B	P01024	-	-0.29	0.73	1	Wahlen [44]
Complement C1r subcomponent	C1R	P00736	-	-0.29	0.74	1	Wahlen [44]

*, pcorr is presented and is the multivariate correlation coefficient of each variable (protein) for the model, with FMS being coded as 1, and HC as 0. Hence, a positive p(corr) for a variable indicates a positive multivariate correlation versus FMS belonging, and a negative p(corr), indicates a negative multivariate correlation with FMS.

**, A VIP > 1 is indicative of a significant protein.

CD14, cluster of differentiation 14 molecule; FMS, fibromyalgia syndrome; pcorr, partial and semipartial correlation coefficients; UniProt, Universal Protein Resource; VIP, variable importance in projection.

Supplementary Table 9. Significance (*p*-value), protein levels, VIP, MS abundance, and fold change in proteins observed in serum samples of patients with FMS against controls.

Protein name	Abbreviation	UniProt	*p*-value	NPX*		VIP	MS AB		Fold change	Reference
				FMS	controls		FMS	controls		
STAM-binding protein	STAMBP	O95630	0.010	4.76	4.09	-	-	-	-	Fineschi [37]
Axin-1	AXIN1	O15169	0.010	3.12	2.09	-	-	-	-	Fineschi [37]
NAD-dependent protein deacetylase sirtuin-2	SIRT2	Q8IXJ6	0.010	4.39	3.43	-	-	-	-	Fineschi [37]
Sulfotransferase 1A1	SULT1A1	P50225	0.031	4.32	3.21	-	-	-	-	Fineschi [37]
Stromelysin-2	MMP-10	P09238	0.044	9.96	9.51	-	-	-	-	Fineschi [37]
Fibroblast growth factor 23	FGF-23	Q9GZV9	0.044	1.83	1.64	-	-	-	-	Fineschi [37]
IL-18 receptor 1	IL-18R1	Q13478	0.044	9.17	8.82	-	-	-	-	Fineschi [37]
TNF ligand superfamily member 14	TNFSF14	O43557	0.044	7.47	6.93	-	-	-	-	Fineschi [37]
Fibroblast growth factor 21	FGF-21	Q9NSA1	0.044	5.13	4.18	-	-	-	-	Fineschi [37]
TNF	TNF	P01375	0.044	3.32	2.96	-	-	-	-	Fineschi [37]
T-cell surface glycoprotein CD5	CD5	P06127	0.048	6.07	5.82	-	-	-	-	Fineschi [37]
TNF receptor superfamily member 5	CD40	P25942	0.048	12.05	11.73	-	-	-	-	Fineschi [37]
C-C motif chemokine 3	CCL3	P10147	0.048	6.99	6.53	-	-	-	-	Fineschi [37]
IL-10 receptor subunit β	IL-10RB	Q08334	0.048	6.76	6.54	-	-	-	-	Fineschi [37]
Fibroblast growth factor 19	FGF-19	O95750	0.048	9.51	8.78		-	-	-	Fineschi [37]
TNF receptor superfamily member 9	TNFRSF9	Q07011	0.048	7.31	6.97	-	-	-	-	Fineschi [37]
IL-8	IL8	P10145	0.048	7.30	6.83	-	-	-	-	Fineschi [37]
Protein S100-A12	EN-RAGE	P80511	0.048	5.99	5.25	-	-	-	-	Fineschi [37]
Macrophage colony-stimulating factor 1	CSF-1	P09603	0.049	10.86	10.72	-	-	-	-	Fineschi [37]
Hornerin	HRNR	Q86YZ3	<0.01	-	-	0.63	1.026±1.485	0.502±0.173	+2.04	Han [46]
Histidine protein methyltransferase 1 homolog	HMT1	O95568	<0.005	-	-	1.69	1.055±0.219	0.660±0.175	+1.60	Han [46]

Protein name	Abbreviation	UniProt	*p*-value	NPX*		VIP	MS AB		Fold change	Reference
				FMS	controls		FMS	controls		
Keratin, type II cytoskeletal 80	KRT80	Q6KB66	<0.05	-	-	0.57	1.112±1.067	0.749±0.209	+1.49	Han [46]
Serum amyloid P-component	SAP	P02743	<0.01	-	-	0.88	1.100±0.383	0.788±0.390	+1.40	Han [46]
Complement C4-A	C4A	P0C0L4	<0.05	-	-	0.96	1.129±0.375	0.851±0.354	+1.33	Han [46]
IL-1 receptor accessory protein	IL1RAP	Q9NPH3	<0.005	-	-	1.18	1.128±0.226	0.856±0.240	+1.32	Han [46]
Transmembrane protease serine 13	TMPRSS1	Q9BYE2	<0.005	-	-	1.61	1.096±0.159	0.841±0.118	+1.30	Han [46]
Low affinity Ig γ Fc region receptor III-B	CD16B	O75015	<0.05	-	-	0.77	1.246±0.353	0.957±0.473	+1.30	Han [46]
Low affinity Ig γ Fc region receptor III-A	CD16A	P08637	<0.05	-	-	0.77	1.246±0.354	0.957±0.473	+1.30	Han [46]
Serum amyloid A-4 protein	SAA4	P35542	<0.05	-	-	0.97	0.906±0.195	1.181±0.381	−1.30	Han [46]
Ig λ chain V-IV region Hil	GLV3-25	P01717	<0.005	-	-	0.73	0.936±0.991	1.224±0.528	−1.31	Han [46]
Platelet glycoprotein V	GP5	P40197	<0.005	-	-	1.07	0.895±0.258	1.176±0.281	−1.31	Han [46]
Prothrombin	F2	P00734	<0.05	-	-	0.79	0.919±0.331	1.207±0.581	−1.31	Han [46]
Ig heavy chain V-I region HG3	HG3	P01743	<0.05	-	-	0.81	0.856±0.731	1.137±0.655	−1.33	Han [46]
Putative V-set and Ig domain-containing-like-protein	GHV1OR21-1	A6NJS3	<0.05	-	-	0.82	0.850±0.732	1.150±0.695	−1.35	Han [46]
Ig heavy chain V-I region V35	GHV1OR15-1	P23083	<0.05	-	-	0.82	0.850±0.732	1.150±0.695	−1.35	Han [46]
Ig λ-2 chain C regions	GLC2	P0CG05	<0.05	-	-	0.76	0.862±0.609	1.172±0.518	−1.36	Han [46]
Thrombospondin-2	THBS2	P35442	<0.005	-	-	1.13	0.898±0.295	1.232±0.346	−1.37	Han [46]
Fibrinogen α chain	FGA	P02671	<0.05	-	-	0.81	0.867±0.490	1.201±0.464	−1.39	Han [46]
Platelet glycoprotein Ib α chain	GP1BA	P07359	<0.01	-	-	1.29	0.847±0.169	1.193±0.333	−1.41	Han [46]
Profilin-1	PFN1	P07737	<0.01	-	-	1.07	0.900±0.205	1.336±0.563	−1.49	Han [46]
Thrombospondin-1	THBS1	P07996	<0.01	-	-	1.01	0.786±0.396	1.250±0.558	−1.59	Han [46]
α1-antitrypsin	AAT	P01009	3.228e-005	-	-	-	-	-	+2	Ruggiero [43]
Transthyretin	TTR	P02766	0.007	-	-	-	-	-	+1.3	Ruggiero [43]

Protein name	Abbreviation	UniProt	*p*-value	NPX*		VIP	MS AB		Fold change	Reference
				FMS	controls		FMS	controls		
Retinol binding protein 4	RBP4	P02753	0.039	-	-	-	-	-	+1.3	Ruggiero [43]
C1qC chain	C1qC	P02747	-	-	-	1.92	-	-	-	Hsu [45]
Protein S100-A7	S100A7	P31151	-	-	-	2.32	-	-	-	Hsu [45]
Serpin B3	SEPRINB3	P29508	-	-	-	2.23	-	-	-	Hsu [45]
Galectin 7	Gal-7	P47929	-	-	-	4.09	-	-	-	Hsu [45]
Lymphatic vessel endothelial hyaluronan receptor 1	LYVE1	Q9Y5Y7	-	-	-	1.67	-	-	-	Hsu [45]
Fibrinogen α chain	FGA	P02671	-	-	-	3.22	-	-	-	Hsu [45]
Fibrinogen β chain	FGB	P02675	-	-	-	4.29	-	-	-	Hsu [45]
Fibrinogen γ chain	FGG	P02679	-	-	-	4.38	-	-	-	Hsu [45]

*, Protein levels expressed as NPX values (log2 scale); CD5, cluster of differentiation 5 molecule; FMS, fibromyalgia syndrome; Ig: Immunoglobulin; IL: Interleukin; MS AB, Mass spectrometry abundance; NAD, Nicotinamide adenine dinucleotide; NPX, normalized protein expression; TNF, Tumor necrosis factor; UniProt, Universal Protein Resource.
